# Hepatocellular glycogenotic foci after combined intraportal pancreatic islet transplantation and knockout of the carbohydrate responsive element binding protein in diabetic mice

**DOI:** 10.18632/oncotarget.22234

**Published:** 2017-11-01

**Authors:** Silvia Ribback, Jenny Sonke, Andrea Lohr, Josephine Frohme, Kristin Peters, Johannes Holm, Michele Peters, Antonio Cigliano, Diego F. Calvisi, Frank Dombrowski

**Affiliations:** ^1^ Institut für Pathologie, Universitaetsmedizin Greifswald, Greifswald, Germany

**Keywords:** preneoplastic foci, hepatocarcinogenesis, intraportal pancreatic islet transplantation, clear cell foci of altered hepatocytes, AKT/mTOR

## Abstract

**Aims:**

The intraportal pancreatic islet transplantation (IPIT) model of diabetic rats is an insulin mediated model of hepatocarcinogenesis characterized by the induction of clear cell foci (CCF) of altered hepatocytes, which are pre-neoplastic lesions excessively storing glycogen (glycogenosis) and exhibiting activation of the AKT/mTOR protooncogenic pathway. In this study, we transferred the IPIT model to the mouse and combined it with the knockout of the transcription factor carbohydrate responsive element binding protein (chREBP).

**Methods:**

C57BL/6J Wild-type (WT) and chREBP-knockout (chREBP-KO) mice (n = 297) were matched to 16 groups (WT/ chREBP-KO, experimental/control, streptozotocine-induced diabetic/not diabetic, one/four weeks). Experimental groups received the intraportal transplantation of 70 pancreatic islets. Liver and pancreatic tissue was examined using histology, morphometry, enzyme- and immunohistochemistry and electron microscopy.

**Results:**

CCF emerged in the liver acini downstream of the transplanted islets. In comparison to WT lesions, CCF of chREBP-KO mice displayed more glycogen accumulation, reduced activity of the gluconeogenic enzyme glucose-6-phosphatase, decreased glycolysis, lipogenesis and reduced levels of the AKT/mTOR cascade members. Proliferative activity of CCF was ∼two folds higher in WT mice than in chREBP-KO mice.

**Conclusions:**

The IPIT model is applicable to mice, as murine CCF resemble preneoplastic liver lesions from this hepatocarcinogenesis model in the rat in terms of morphological, metabolic and molecular alterations and proliferative activity, which is diminished after chREBP knockout. chREBP appears to be an essential component of AKT/mTOR mediated cell proliferation and the metabolic switch from a glycogenotic to lipogenic phenotype in precursor lesions of hepatocarcinogenesis.

## INTRODUCTION

### Pre-neoplastic hepatocellular lesions in rodent models and humans

Hepatocellular carcinoma (HCC) is one of the most frequent neoplasms and its incidence is rapidly rising in Western Europe and the United States [1]. In particular, it has been shown that 15-20% of HCC occur in the non-cirrhotic liver [2]. Besides classical risk factors for liver cirrhosis, epidemiologic studies indicate that acquired metabolic diseases like adiposity, hyperinsulinism and type 2 diabetes mellitus are implicated in HCC development as well [3, 4]. These cases occur often in the non-cirrhotic liver in the context of non-alcoholic steatohepatitis, whose potential role in hepatocarcinogenesis has been recently investigated [4-8]. Until today, human HCC development without preexisting cirrhosis is not well understood. In animal models of hepatocarcinogenesis, preneoplastic liver lesions have been identified and may proceed to hepatocellular adenomas (HCA) and HCC without a background of liver cirrhosis [9-13]. Among them, clear cell foci (CCF) are the most common. CCF are well characterized glycogen storing lesions and reveal alterations of glucose and lipid metabolism, which resemble insulin effects on hepatocytes. Indeed, they can also be induced after intraportal transplantation of pancreatic islets at the downstream level in the hepatocellular acinus in diabetic rats. In longterm investigations, these CCF proceed to HCA and HCC [14-17]. So far, the intrahepatic pancreatic islet transplantation (IPIT) model of hepatocarcinogenesis has been exclusively established in the rat. In humans, CCF are known to occur both in the cirrhotic [18, 19] and non-cirrhotic [18, 20] liver. Human CCF display morphologic, metabolic, and molecular alterations similar to those of pre-neoplastic CCF induced in rat models of hepatocarcinogenesis by local hyperinsulinism [15, 21], insulinomimetic effects of chemicals [11], or viruses [10, 12, 13]. Disturbed glycogen breakdown results in the glycogenic phenotype [11], which is often accompanied by *de novo* lipogenesis [22] leading to the lypogenic phenotype [20, 23-25]. These changes are associated with a reduction in gluconeogenesis, an increasing channeling of glucose-6-phosphate into glycolysis and the pentose phosphate pathway, activation of the phosphoinositide-3-kinase/V-akt murine thymoma viral oncogene homolog/mammalian target of Rapamycin (PI3K/AKT/mTOR) and Rat sarcoma/mitogen activated protein kinase (Ras/MAPK) pathways, which are downstream effectors of insulin [20-23]. Activation of these signaling cascades and the lipogenic phenotype are also typical findings in human HCC, and associated with a worse prognosis [24, 25]. These findings suggest that CCF are very early pre-neoplastic lesions with low-propensity to evolve into HCC in humans [20].

### Role of the carbohydrate responsive element binding protein (chREBP) in hepatocellular metabolism and molecular hepatocarcinogenesis

Activation of the AKT/mTOR cascade and alterations of glucose and lipid metabolism in CCF and HCC of humans and rats are accompanied by the upregulation of *carbohydrate responsive element binding protein* (chREBP) transcription factor [20, 23]. Recently, chREBP has been highlighted in the genesis of the metabolic syndrome in experimental mouse models [26-29]. It is activated dependently and independently of insulin during long lasting hyperglycemia, requiring generation of glucose-6-phoshate by the glucokinase [30] and binds to the *carbohydrate responsive element* (ChoRE) in the promoter region of the pyruvate kinase (liver type). Target proteins of chREBP are involved in glycolysis, lipogenesis and gluconeogenesis. chREBP is, together with the *sterol regulatory element binding protein-1c* (SREBP-1c), a major regulator of hepatocellular glucose and lipid metabolism [29]. Due to its role as a regulator of glucose- and lipid metabolism and suggestive mediator of proto-oncogenic signaling via AKT/mTOR, chREBP is an appropriate target for a mouse-knockout-model to characterize the association of impaired metabolism and molecular alterations in the hepatocarcinogenesis process. Therefore, we transferred the pancreatic islet transplantation model to the mouse and combined it with the knockout of chREBP.

## RESULTS

### Body weight and blood glucose level

chREBP-KO mice displayed an elevated body weight in comparison to WT mice in almost all experimental groups (Supplementary Figure 1). The diabetic groups revealed an expected weight loss in comparison to non-diabetic mice. Weight loss of transplanted, diabetic WT mice was lower than in diabetic, not transplanted mice. As intended, transplanted diabetic mice remained hyperglycemic, due to the paucity of transplanted islets. Continuous hyperglycemia is necessary in this model, so that the islets are constantly stimulated for permanent maximal insulin synthesis and secretion. As a result, local hyperinsulinism and simultaneous hyperglycemia is obtained at the downstream liver acini.

Blood glucose level was elevated in diabetic groups in comparison to non-diabetic mice, with only slight differences between genotypes (Supplementary Figure 1).

### Morphology and proliferative activity of β-cells in “in-situ”- intrapancreatic islets

In general, the β-cell proportion in intrapancreatic islets of diabetic mice was about three times lower (range 6-23%) than in non-diabetic control mice (56-74%) *(data not shown)* due to streptozotocine toxicity, without differences between genotypes.

There were no signs of necrosis or inflammation. BrdU-LI of β-cells was higher in diabetic groups due to the stimulation of cell proliferation by hyperglycemia. There was no difference between genotypes in the transplanted groups. Diabetic, not transplanted WT mice revealed a significantly higher BrdU-LI than diabetic transplanted WT mice (after one week) and also in comparison to the corresponding chREBP-KO group (after one and four weeks) (Table 1, Supplementary Figure 2).

**Table 1:** Proliferative activity of pancreatic islets within the pancreas of C57B6/J wildtype (WT) and chREBP-knockout (chREBP-KO) mice

	WT	chREBP-KO
**Groups**	**ß-cells** **BrdU-LI %** **mean±S.E.M. (n)**	**ß-cells** **BrdU-LI %** **mean±S.E.M. (n)**
diabetic transplantation 1 week	**3.85 ± 1.18 (10)** ^*,#^	**2.99 ± 0.44 (9)**
diabetic control 1 week	**12.17 ± 2.59 (9)** ^*,#, **§**^	**1.03 ± 0.59 (9)** ^**§**^
diabetic transplantation 4 weeks	**3.66 ± 0.72 (10)** ^#^	**4.04 ± 0.91 (10)** ^*^
diabetic control 4 weeks	**4.09 ± 0.42 (8)** ^#, **§**^	**1.40 ± 0.52 (6)** ^*, §^
non diabetic control 1 week	**0.90 ± 0.79 (8)**	**0.61 ± 0.09 (8)**
non diabetic control 4 weeks	**0.47 ± 0.17 (10)**	**0.28 ± 0.12 (10)**

Proliferative activity (BrdU-labeling index/LI) of pancreatic β-cells after BrdU application with an osmotic minipump for 1 week. S.E.M. standard error of the mean. comparisons: ^*^ diabetic and transplantation vs. diabetic and control; ^#^ diabetic and transplantation/ diabetic control vs. non diabetic control; ^§^ WT vs. chREBP-KO, p < 0.05.

### Morphology, glycogen content, and enzyme activity of liver parenchyma in non-diabetic and diabetic WT and chREBP-KO mice

#### Non-diabetic mice

Non-diabetic WT mice livers revealed regular glycogen content and distribution of the glucose-6-phosphatase (G6Pase) and glucose-6-phosphate-dehydrogenase (G6PDH) enzymes, with a gradient of activity from acinar zone 1 to zone 3. In comparison, hepatocytes of non-diabetic chREBP-KO mice contained slightly more glycogen, less mitochondria and lower activity of G6Pase and G6PDH than WT mice livers (Supplementary Figures 3 and 7).

#### Diabetic mice

Hepatocytes of diabetic WT mice contained sparse glycogen and showed high activity of the G6Pase, a slight reduction of G6PDH activity, and an increase and enlargement of mitochondria and the endoplasmatic reticulum in comparison to non-diabetic mice. On the contrary, diabetic chREBP-KO mouse livers exhibited a retained glycogen content due to apparent reduction of G6Pase activity and an elevated G6PDH activity in acinus zone 1 and also enlarged mitochondria (Supplementary Figures 3 and 7).

### Morphological alterations of hepatocytes after intraportal pancreatic islet transplantation in diabetic mice

#### WT mice

**Figure 1:**
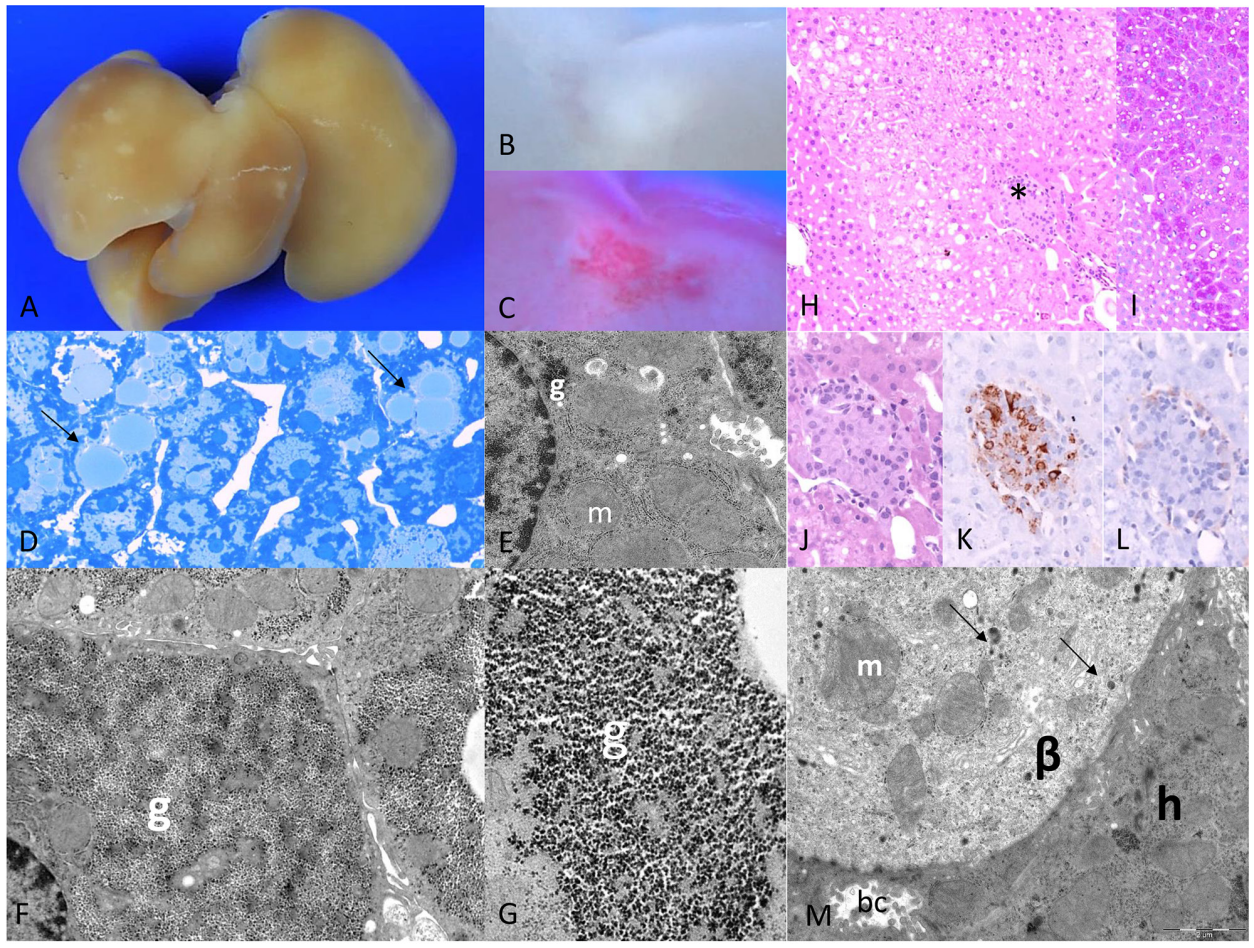
Diabetic WT mice one week after intraportal pancreatic islet transplantation **(A)** Liver surface with multiple small pale lesions (after perfusion fixation the blood was washed out), and **(B)** corresponding cut surface **(C)** lipid storage within lesions in Sudan red staining, **(D)** corresponding to lipid droplets in semithin section (arrows); **(E)** ultrastructural micrograph of extrafocal hepatocyte with reduced glycogen (g) and enlarged mitochondria (m); **(F)** altered hepatocyte in CCF with increase of glycogen storage replacing mitochondria beneath the nucleus consisting of **(G)** α-glycogen particles (g); **(H)** acinar organized clear cell alterations of hepatocytes at the downstream level of a transplanted islet (^*^, H&E), **(I)** increased glycogen storage of hepatocytes depicted in PAS-reaction, **(J)** higher magnification (H&E), **(K)** β-cells educible in the insulin immunostaining, **(L)** few α-cells in glucagon immunostaining, **(M)** ultrastructure of transplanted islet with activated, degranulated β-cell (β) with few granules (arrows) and enlarged mitochondria (m), neighboring a hepatocyte (h) with a bile canaliculus (bc). Length of the lower edge: A 1,5 cm; B, C 3mm; D 0.08mm; E 6.4μm; F 9μm; G 2μm; H, I 0.4mm; J, K, L 0.19 mm; M 12μm.

**Figure 2:**
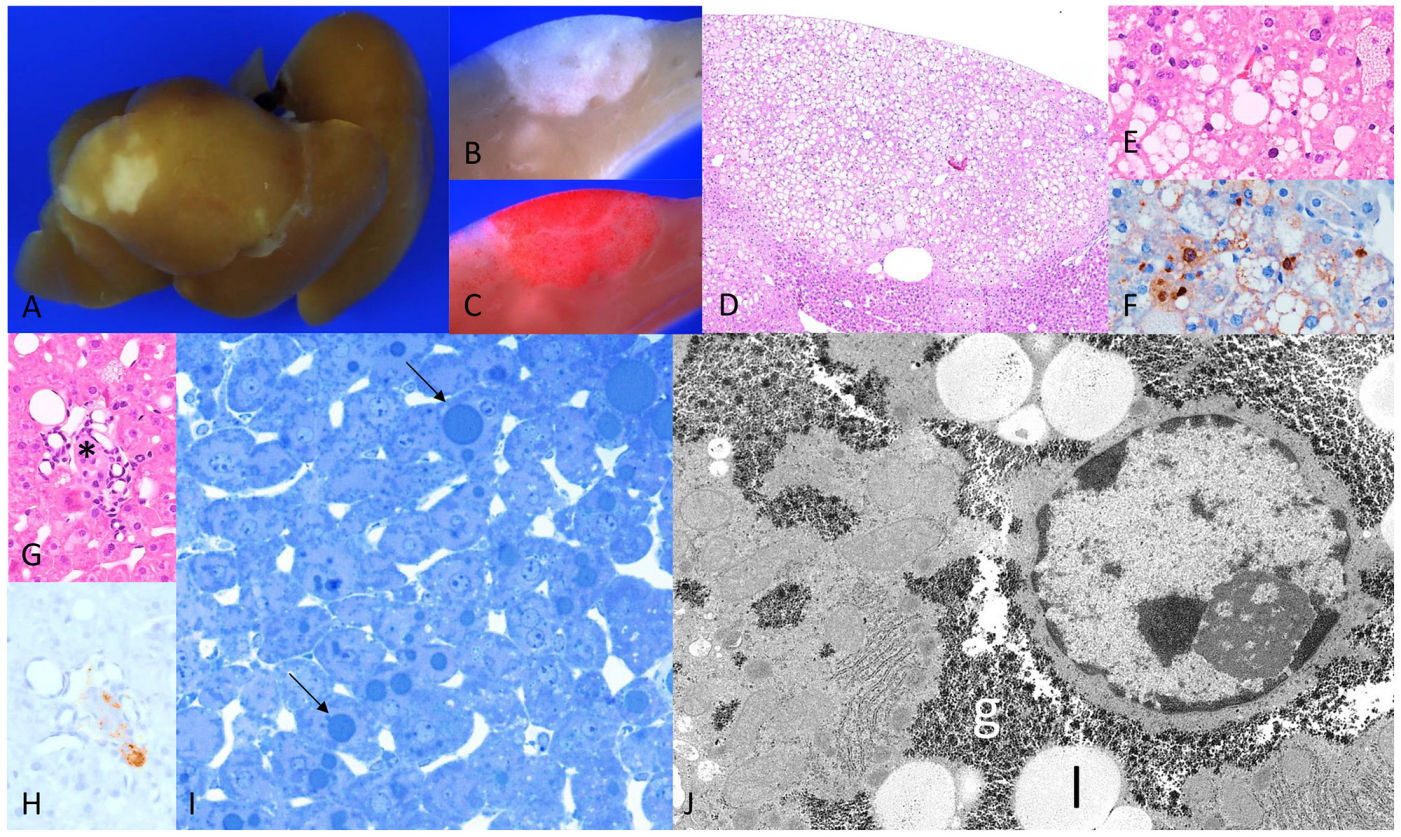
Diabetic WT mice four weeks after intraportal pancreatic islet transplantation **(A)** Liver surface with larger lesions, and **(B)** corresponding cut surface **(C)** increased lipid storage in Sudan red staining, **(D)** corresponding H&E with **(E)** fat vacuoles and **(F)** proliferating hepatocytes in BrdU immunostaining; **(G)** transplanted islet (^*^, H&E) with **(H)** β-cells in insulin immunostaining **(I)** semithin section hepatocytes with lipid droplets (arrows) **(J)** hepatocyte within CCF with increased glycogen (g) storage and lipid vacuole (l). Length of the lower edge: A 1.5cm; B, C 4mm; D 2,5mm; E, F 0.5mm; G, H 0.25mm; I 0.13 mm; J 16 μm.

After one week, multiple small pale lesions were visible on the liver surface, corresponding to CCF in the liver acini downstream of the pancreatic islet transplants in peripheral portal vein branches (Figure 1). Hepatocytes revealed a clear cell morphology in H&E staining, corresponding to an increased storage of glycogen with purple PAS reaction and lipid storage with red droplets in Sudan red staining as a consequence of the local hyperinsulinism and simultaneous hyperglycemia, whereas extrafocal liver parenchyma comprised sparse glycogen (Supplementary Figure 3). Ultrastructural analysis of CCF confirmed increased glycogen storage as α-particles and lipid droplets in the cytoplasm (Figure 1), in comparison to extrafocal hepatocytes with a glycogen-poor cytoplasm (Supplementary Figure 3). β-cells of transplanted islets were activated, as indicated by enlarged mitochondria and degranulated vesicles (Figure 1). After four weeks, clear cell lesions extended and often occupied several liver acini (Figure 2), without architecture disruption. Proliferative activity (BrdU-LI) was increased in hepatocytes of CCF in comparison to extrafocal liver parenchyma after four weeks (Table 2).

**Table 2:** Proliferative activity of liver clear cell foci and extrafocal liver parenchyma in C57B6/J wildtype (WT) and chREBP-knockout (chREBP-KO) mice

(A)		
Experimental groups with transplantation	Clear cell foci BrdU-LI % mean±S.E.M. (n)	Extrafocal liver BrdU-LI % mean±S.E.M. (n)
**WT** Diabetic and transplantation 1 week	3.03 ± 1.39 (8)	0.62 ± 0.31(8)
**chREBP-KO** diabetic and transplantation 1 week	1.33 ± 0.25 (9)^*^	0.60 ± 0.14 (9)^*^
**WT** Diabetic and transplantation 4 weeks	11.34 ± 2.24(9)^*,#^	2.40 ± 0.71(9)^*^
**chREBP-KO** diabetic and transplantation 4 weeks	6.08 ± 0.70(12)^*,#^	1.91 ± 0.20(12)^*^
**WT** non diabetic and transplantation 1 week	-	1.21 ± 0.02(3)
**chREBP-KO** non diabetic and transplantation 1 week	-	0.80 ± 0.26(6)
**WT** non diabetic and transplantation 4 weeks	-	4.03 ± 0.41(4) ^#^
**chREBP-KO** non diabetic and transplantation 4 weeks	-	0.93 ± 0.14(5) ^#^

Proliferative activity (BrdU-labeling index/LI) of liver clear cell foci and extrafocal liver parenchyma after BrdU application with an osmotic minipump 1 week. S.E.M. standard error of the mean., comparisons: ^*^ clear cell foci vs. unaltered liver tissue; ^#^ WT vs. chREBP-KO. p < 0.05.

**Table:** 

(B)	
Control groups without transplantation	Extrafocal liver BrdU-LI % mean±S.E.M. (n)
**WT** diabetic 1 week	0.26 ± 0.06(8) ^#^
**chREBP-KO** diabetic 1 week	0.07 ± 0.03(5) ^#^
**WT** diabetic 4 weeks	0.31 ± 0.09(5) ^#^
**chREBP-KO** diabetic 4 weeks	0.02 ± 0.02(5) ^#^
**WT** non diabetic 1 week	0.99 ± 0.30(8)
**chREBP-KO** not diabetic 1 week	0.26 ± 0.05(5)
**WT** non diabetic 4 weeks	0.93 ± 0.18(5)
**chREBP-KO** non diabetic 4 weeks	0.38 ± 0.18(5)

Proliferative activity (BrdU-labeling index/LI) of liver clear cell foci and unaltered liver parenchyma after BrdU application with an osmotic minipump 1 week. S.E.M. standard error of the mean., comparisons: ^*^ clear cell foci vs. unaltered liver tissue; ^#^ WT vs. chREBP-KO. p < 0.05.

#### chREBP-KO mice

**Figure 3:**
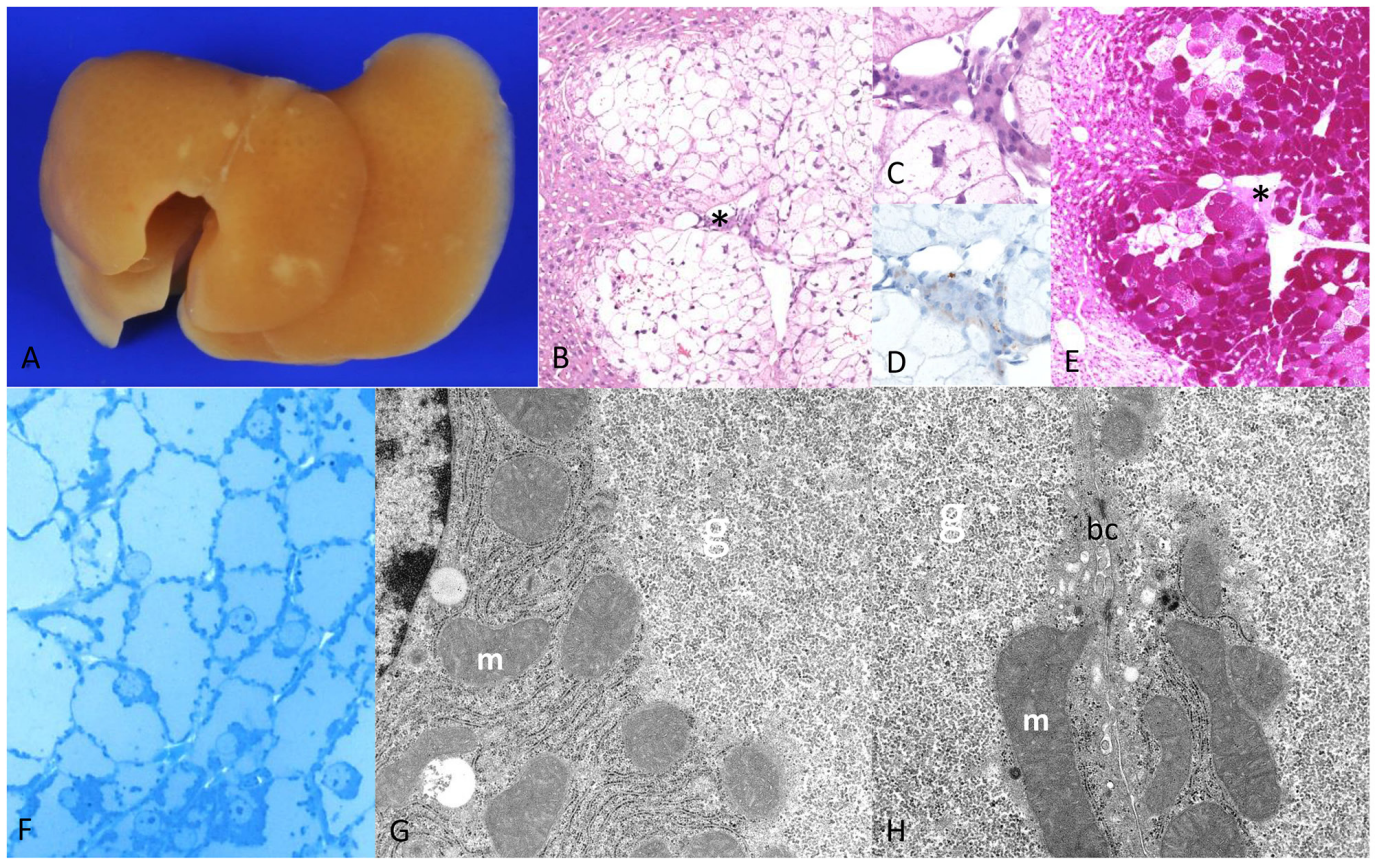
Diabetic chREBP-KO mice one week after intraportal pancreatic islet transplantation **(A)** Liver surface with multiple small white-yellow lesions like in WT mice (after perfusion fixation the blood was washed out) with **(B)** altered hepatocytes with clear cell alteration downstream of a transplanted islet (^*^, **(C)** magnification, **(D)** - highlighted β-cells in insulin immunostaining) **(E)** deep purple cytoplasm in the PAS reaction due to massive glycogen storage **(F)** semithin section of a CCF with enlarged hepatocytes with pale homogenous cytoplasm due to **(G, H)** masses of α-glycogen particles (g) displacing mitochondria (m) to the outer cell borders (bc - bile canaliculus between two hepatocytes). Note the lack of lipid vacuoles. Length of the lower edge: A 1.5cm; B 6mm; C, D 0.08mm; E 6mm; F 0.16mm; G, H 7.6μm.

**Figure 4:**
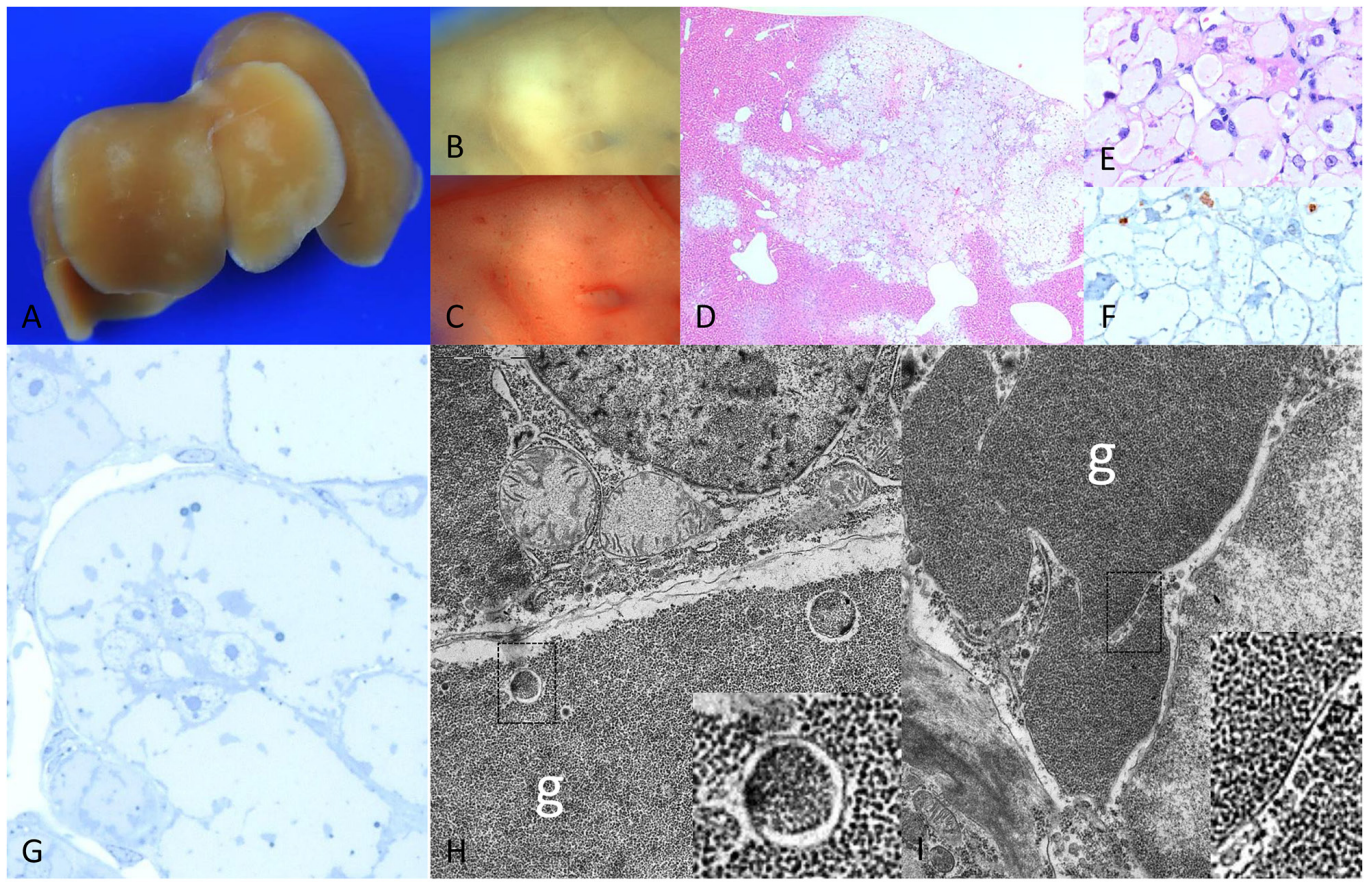
Diabetic chREBP-KO mice 4 weeks after intraportal pancreatic islet transplantation **(A)** Liver surface with multiple pale lesions **(B)** corresponding cut surface **(C)** lack of lipids in Sudan red staining **(D)** corresponding H&E staining with **(E)** enlarged hepatocytes with clear cell morphology lacking lipid droplets and **(F)** low proliferative activity in BrdU immunostaining **(G)** semithin section with broad pale cytoplasm of altered hepatocytes **(H, I)** ultrastructural micrographs of altered hepatocyte with masses of glycogen particles (g), often segregated by membrane-like structures (higher magnifications in squares). Length of the lower edge: A 1.5cm; B, C, D 3mm; E, F 0.16mm; G 0.08mm; H, I 8.8 μm.

Livers of chREBP-KO mice also revealed several pale lesions of about 1mm in size on the liver surface, consisting of CCF in the liver acini downstream of transplanted islets with increased BrdU-LI in comparison to extrafocal liver parenchyma after one and four weeks (Table 2). However, morphology of these hepatocytes differed from WT lesions. Indeed, hepatocytes were significantly larger and contained much more glycogen, and lipid storage was significantly less than in WT mice (Figure 3). Interestingly, extrafocal liver parenchyma still contained glycogen despite hypoinsulinism occurred (Supplementary Figure 3). Hepatocytes of CCF were often associated with slight inflammatory changes with surrounding leukocytes (Supplementary Figure 10). At the ultrastructural level, cytoplasm of CCF was occupied by masses of predominantly free glycogen α-particles displacing other cell organelles to the outer cell borders. Furthermore, glycogen was segregated in vesicles and vacuoles, most likely representing autophagic vacuoles (Figure 4).

#### Incidence of CCF in WT and chREBP-KO mice

CCF emerged more frequently in chREBP-KO than in WT mice (WT vs. chREBP-KO one week 67% vs. 95%; four weeks 45% vs. 82%, as % of transplanted mice revealing CCF, p < 0.05, Supplementary Figure 4). Regarding low incidence of CCF in WT mice, we excluded early transplant failure by demonstrating viability of isolated islets (Supplementary Figure 5). Of note, by incrementing the number of transplanted islets to 120 and 200 or preoperative insulin treatment, respectively, incidence of CCF in WT mice could not be increased. Blood glucose level was significantly lowered in the first week after islet transplantation in WT mice (Supplementary Figure 6), suggesting initial engraftment, but normoglycemia was not reached. Nevertheless, after 2 weeks blood glucose rised progressively, suggesting loss of intraportal islets or exhaustion of insulin secretion of the β-cells.

#### Comparison of proliferative activity of hepatocytes in WT and chREBP-KO mice

**Figure 5:**
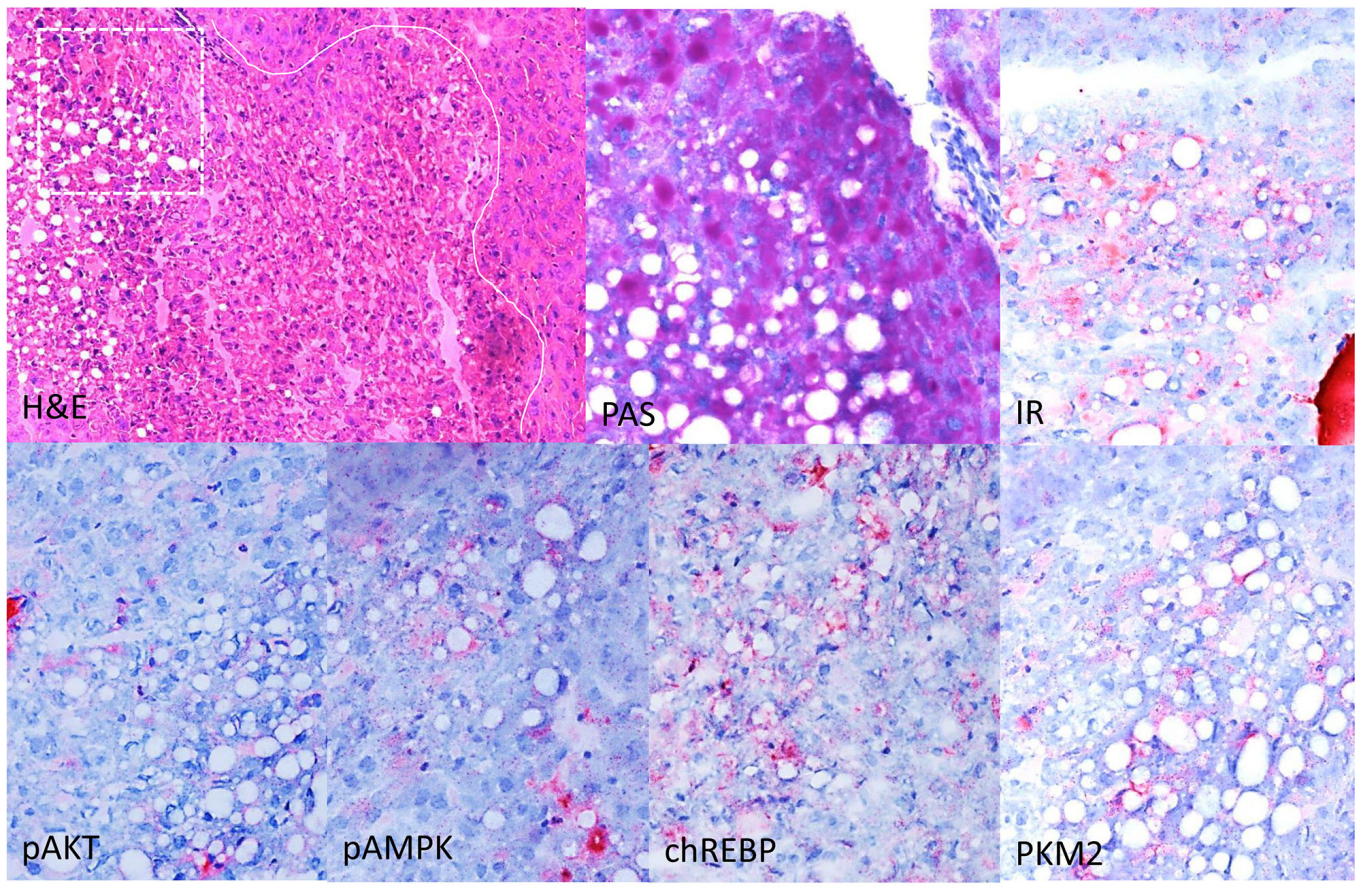
Immunohistochemical characterization of CCF in the liver of diabetic WT mice, one week after intraportal pancreatic islet transplantation Focus of altered hepatocytes (white line as border to neighboring extrafocal liver tissue) with clear cell morphology (H&E), glycogen storage and lipid droplets in the PAS reaction. Altered hepatocytes display a stronger immunoreactivity for the insulin receptor (IR), activated AKT and AMP-Kinase (p-AKT, pAMPK), the transcription factor chREBP and upregulation of glycolysis (pyruvate kinase M2 (PKM2). Serial frozen sections. Length of the lower edge: H&E 0.75mm; magnified square (PAS and immunostainings) 0.15mm.

Proliferative activity in CCF of KO mice was half than that in WT mice after four weeks *(*BrdU-LI of CCF, WT vs. chREBP-KO after four weeks: 11.34% ± 2.24% vs. 6.08% ± 0.70%; p < 0.05, Table 2A, Figure 5).

BrdU-LI of extrafocal hepatocytes was lower in KO than in WT mice in three groups, namely in diabetic mice without transplantation (after one and four weeks) and transplanted non-diabetic mice after four weeks (Table 2A and 2B).

#### Enzyme histochemistry in WT and chREBP-KO mice

Control liver parenchyma of non-diabetic WT mice reflected the well known metabolic zonation showing gradients in enzyme activities from zone 1 to zone 3 of the acini for the histochemical assays of G6Pase and G6PDH. Non-diabetic chREBP-KO liver parenchyma was characterized by slightly higher glycogen content and was associated with lower activity of the glyconeogenic enzyme G6Pase and the key enzyme of the pentose phosphate pathway G6PDH (Supplementary Figure 7).

Liver tissue of diabetic WT mice showed loss of glycogen storage and an intense increase of G6Pase activity and a reduction of G6PDH activity due to extrafocal hypoinsulinism, whereas diabetic chREBP-KO mice retained glycogen and demonstrated a distinct reduction of G6Pase activity. G6PDH activity was elevated in acinus zone 1 (Supplementary Figure 7).

One week after intraportal islet transplantation, G6Pase activity was reduced in glycogen storing CCF of diabetic WT mice and almost completely lost in CCF of KO mice in comparison to the respective extrafocal liver parenchyma, attributable to the local hyperinsulinism produced by the transplanted islets. On the contrary, G6PDH activity was upregulated in CCF of WT mice, but almost inactive in CCF of chREBP-KO mice, a most remarcable finding (Supplementary Figure 8).

### Immunohistochemical characterisation

Cyclin D1 was overexpressed in CCF in comparison to extrafocal liver tissue in CCF of diabetic WT and chREBP-KO mice, corresponding to an activated cell cycle. Nevertheless, Cyclin D1 protein levels did not differ between genotypes. Cyclin dependent kinase 4 (CDK4) was not induced (Supplementary Figure 9).

As expected, CCF of diabetic WT mice showed an upregulation of the insulin receptor in comparison to the surrounding liver parenchyma, and protooncogenic AKT/mTOR signaling was activated including an overexpression of activated (phsophorylated) AKT (p-AKT). Furthermore, the transcription factor chREBP and glycolysis mediator pyruvate kinase M2 (PKM2) were upregulated (Figure 5).

**Figure 6:**
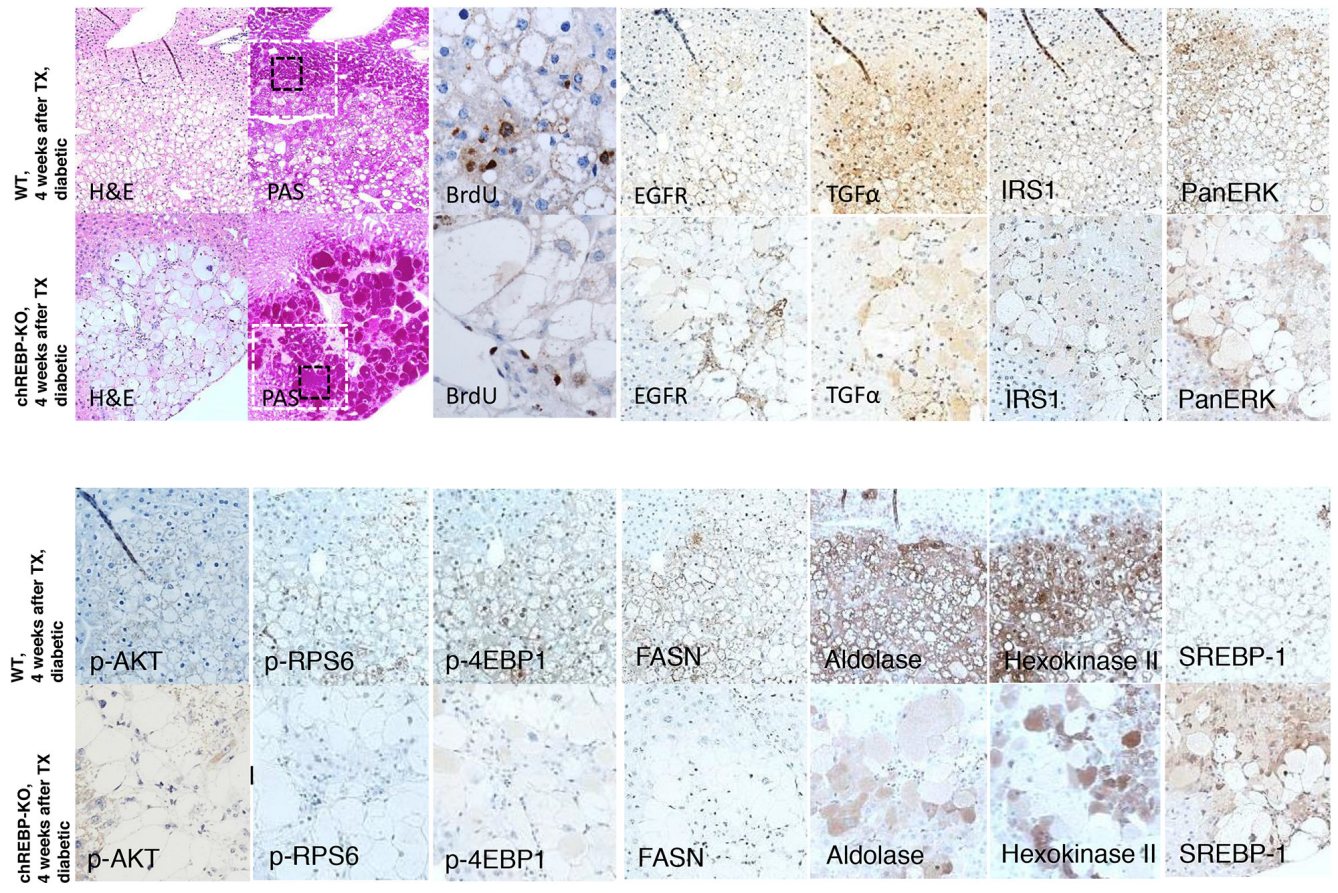
Comparison of hepatocellular CCF of diabetic WT mice and diabetic chREBP-KO mice, four weeks after intraportal pancreatic islet transplantation (TX) Foci of altered hepatocytes with clear cell morphology (H&E) in chREBP-KO mice exhibit a much more pronounced glycogen storage (PAS), and a lower proliferative activity (BrdU, corresponding magnification of black square) than lesions of WT mice. In CCF of WT mice, the epidermal growth factor receptor (EGFR), its ligand transforming growth factor α (TGFα), protooncogenic cascades of ras/raf-1 (IRS1, PanERK) and AKT/mTOR (p-AKT, p-RPS6, p4EBP-1) and de-novo-lipogenesis with fatty acid synthase (FASN) and glycolysis with aldolase and hexokinase II are activated. On the contrary, these pathways and metabolic alterations are less strongly upregulated in chREBP-KO lesions. By contrast, the transcription factor sterol regulatory element-binding protein 1 (SREBP-1) is higher expressed in CCF of chREBP-KO than WT lesions. Serial paraffin sections. Length of the lower edge: H&E, PAS 0.6mm; black square - BrdU 0.13mm; white square - immunostainings 0.4mm.

Comparison of CCF in WT and chREBP-KO mice revealed a stronger overexpression of the epidermal growth factor receptor (EGFR) and its ligand, transforming growth factor α (TGFα), and a more pronounced activation of the protooncogenic cascades downstream of Ras/raf-1/MAPK- (IRS1, PanERK) and AKT/mTOR-signaling pathways (p-AKT, p-RPS6, p4EBP-1) in WT than in chREBP-KO mice. In addition, glycolysis (aldolase A and hexokinase II) and de-novo-lipogenesis with fatty acid synthase (FASN) were overexpressed in CCF of WT mice, but significantly less in KO lesions, thus well corresponding to the lack of lipid storage. By contrast, the transcription factor sterol regulatory element-binding protein 1 (SREBP-1) was more strikingly induced in CCF of chREBP-KO than in WT mice (Figure 6).

In CCF of chREBP-KO mice, tumor necrosis factor α (TNFα) and inducible nitric oxide synthase (iNOS) were detectable in inflammatory cells, but also focally in hepatocytes (Supplementary Figure 10), whereas CCF of WT mice did not show an inflammatory reaction.

## DISCUSSION

To the best of our knowledge, this is the first study showing a successful transfer of the intraportal pancreatic islet transplantation model to the mouse with a carcinogenic, and less therapeutical, approach and constitutes the basis for the intended combination of this model with knockout models. In the background of a streptozotocine induced diabetes, the transplanted pancreatic islets engrafted in the portal vein branches, resulting in a local hyperinsulinism in combination with persistent hyperglycemia in the liver acini downstream of the transplanted islets, leading to glycogen and fat accumulation in the liver of WT mice, eliciting the typical glycogen storing foci of altered hepatocytes, presenting as CCF in conventional tissue preparations. In general, the morphological alterations of these murine hepatocellular CCF are attributable to the effects of local hyperinsulinism on hepatocytes downstream of the transplanted islets and resemble preneoplastic liver lesions observed in the rat after IPIT [14-17] or insulinomimetic effects of various oncogenic agents [11] and in other hepatocarcinogenesis mouse models [13, 31]. In addition to the morphologic similarities, our studies on CCF of WT mice substantiated previous findings in rat hepatocarcinogenesis models and human HCC, particulary increased cell proliferation and metabolic alterations like upregulated glycolysis and lipogenesis and activated Ras/raf-1/MAPK and AKT/mTOR protooncogenic signaling pathways, including an overexpression of the transcription factor chREBP [23, 25].

The observation of lower β-cell proliferation in the intrapancreatic islet tissue in diabetic chREBP-KO mice is in line with previous investigations of Metukuri et al. [32], as chREBP also mediates the glucose-stimulated β-cell proliferation and may contribute to glucose toxicity of β-cells [33]. In this context, chREBP seems to play an essential role in the development of glucose intolerance and of insulin resistance in pancreatic β-cells and in hepatocytes [34], which might also be an explanation for the lower incidence of CCF in diabetic WT mice than in chREBP-KO mice in our investigations. We excluded frequent reasons for islet graft failure like thrombosis and inflammation, avitality of transplants [35], and modified the experiment by increasing number of transplants and pre-operative insulin therapy to reduce possible glucotoxictiy [36] in diabetic WT mice. The islets seem to engraft at first, because blood glucose level was significantly reduced and the incidence of CCF was increased after one week. However, after four weeks the incidence of CCF was lower than after one week, suggesting a late graft failure, indicating an insulin resistance in diabetic WT mice, maybe also attributable to chREBP, as it has been suggested by Iizuka [37].

The chREBP knockout led not only to a higher incidence of CCF after IPIT in these KO mice compared to WT mice, but also to additional striking morphological, metabolic and molecular alterations within these lesions and also in the extrafocal hepatocytes. There have been many biochemical and molecular investigations on chREBP-KO mice and on cell cultures derived from these mice [26, 27, 37], but to the best of our knowledge, no histomorphological descriptions CCF emerging in their liver parenchyma have been published to date. Furthermore, the effects of a manifest diabetic state in these mice have not been studied so far.

The most impressive morphological difference between CCF in chREBP-KO mice in comparison to CCF in WT mice was the massive storage of free glycogen in the hepatocellular cytoplasm without an accompanying lipid accumulation. This is in line with biochemical assays conducted in chREBP-KO mice [37, 38]. In particular, the reduction of the activity of the glyconeogenotic enzyme G6Pase and glycolysis is a possible explanation for glycogenosis, as chREBP upregulates hepatic G6Pase and glycolysis proteins (i.e. GLUT2, PKM2) gene expression directly in response to glucose increasing intracellular glucose concentration [37, 38]. chREBP has also been shown to be involved in *de novo* lipogenesis [37]. This apparently explains the lack of lipids in CCF of chREBP-KO mice when compared to WT mice.

The combination of reduced G6Pase and increased activity of G6PDH, the key enzyme of the pentose phosphate shunt, observed in CCF of WT mice has been well documented in insulin induced preneoplastic liver lesions of the rat [14] or insulinomimetic effects of chemicals [11, 39]. Thus, the finding of the combined reduced activity of G6Pase and downregulation of the pentose phosphate pathway with lower activity of G6PDH in CCF of chREBP-KO mice is remarcable, which is in line with the biochemical observations in tissue homogenates of the livers from chREBP-KO mice reported by Iizuka et al. [26, 37]. The low activity of G6PDH in the strongly glycogenotic CCF of chREBP-KO mice correlated with a low proliferative activity and a missing lipid storage. This in accordance with findings in pre-neoplastic liver foci in studies on chemical hepatocarcinogenesis in the rat, which reported an inverse correlation between the accumulation of glycogen and cell proliferation during progression from the well differentiated glycogen storing foci to the de-differentiated rapidly growing, glycogen poor lesions [40]. Furthermore, this transformation process from glycogenotic to glycogen poor lesions is associated with transient lipid accumulation [11] and an increased activity of the G6PDH, suggesting an upregulated pentose phosphate shunt during progression, favoring cell growth and proliferation [39].

Furthermore, the alterations in CCF in KO mice compared to the lesions of WT mice resemble the effects of the tumor suppressor gene p53 in cancer cells [41] and inversely of the protooncogenic Akt/mTOR pathway, suggesting that chREBP suppresses p53 activity and switches oxidative metabolism to aerobic glycolysis, known as the Warburg effect, in cancer cells [42]. Therefore, the additional findings of reduced glycolysis and *de novo*-lipogenesis as downstream effects of the downregulated signaling of AKT/mTOR, the EGFR and Ras/raf-1/MAPK-pathways [20, 22] in CCF of chREBP-KO mice indicate a chREBP mediated metabolic switch also in early hepatocellular clear cell lesions. Nevertheless, a correlation of chREBP mediated p53 inhibition in altered hepatocytes in WT CCF should be verified in future experiments.

Despite the impressive hepatocellular swelling due to glycogenosis, the CCF lesions of chREBP-KO mice revealed a significant lower increase of hepatocellular proliferation in association with impeded lipogenesis, contributing to the growth property of AKT/mTOR signaling [20, 22, 25], and in particular of chREBP mediated gene expression alterations [37]. Nevertheless, the phenomen of intensive glycogen storage with an inverse correlation to cell proliferation is well known in early preneoplastic lesions of other hepatocarcinogenesis models [40], and does not exclude a preneoplastic nature. Thus, a tumor suppressive function of chREBP cannot be excluded.

Membranous segregation of glycogen masses in glycogenotic hepatocytes has been repeatedly reported in the rat [9, 12] and suggests an autophagic process that needs to be clarified in further investigations. This phenomen is probably related to an increase in the activity of the α-glucosidase, hydrolytically breaking down glycogen [43] in lysosomes (so called glycogenosomes).

## MATERIALS AND METHODS

(For detailed methodology please refer to electronic supplementary material)

In this study, all animals received humane care according to the criteria outlined in the “Guide for the Care and Use of Laboratory Animals”, prepared by the National Academy of Sciences and published by the National Institutes of Health (NIH publication 86-23 revised 1985) was followed. Animal experiments were approved by the Animal Policy and Welfare Committee of the Universitaetsmedizin Greifswald, Germany (LALLF-MV Rostock, Germany, ref. no. 7221.3-1.1-003/12 and 7221.3-1-022/14). Housing of the animals was in accordance with the guidelines of the Society for Laboratory Animal Service and the German Animal Protection Law.

Highly inbred 6-weeks-old male C57BL/6J wild-type (WT, CHREBP^+/+^) and chREBP-knockout (chREBP-KO, CHREBP^-/-^) mice (n = 297; 25-30 g body weight) were purchased from Charles River Laboratories (Sulzfeld, Germany). Mice were matched to 16 groups (WT/KO, experimental/control, streptozotocine-induced diabetic/not diabetic, one/ four weeks) as shown in Table 3.

**Table 3:** Experimental and control groups

	Transplantation				Control			
	1 week diabetic	1 week non diabetic	4 weeks diabetic	4 weeks non diabetic	1 week diabetic	1 week non diabetic	4 weeks diabetic	4 weeks Non diabetic
C57Bl/6J (WT)	N= 21	N = 22	N = 18	N = 19	N= 20	N = 21	N = 15	N = 20
chREBP-knockout (chREBP-KO)	N = 20	N= 17	N = 17	N = 17	N = 21	N = 17	N = 15	N = 17

### Genotyping B6.129S6-Mlxipl^tm1Kuy^/J mice

The knockout of the allele Mlxi^pltm1^Kuy (replaced genomic region spanning exons 12-14 [26]) was verified.

### Diabetes induction

Diabetes was induced with a single intraperitoneal dose of streptozotocine (180 mg/kg body weight, [44]). Mice with a blood glucose level > 20 mmol/l after five days were considered diabetic. Transplantation was performed at least after 1 week.

### Transplantation

Recipient WT or chREBP-KO mice, respectively, received an intraportal transplantation of 60-70 isolated, isologous pancreatic islets into the liver under anaesthesia (50-100 mg/kg bodyweight ketamine, 10 mg/kg bodyweight xylazine) via cannulization of the portal vein.

Details of additional modification experiments with higher numbers of transplanted islets and preoperative insulin treatment are explained in Supplementary Materials and Methods and Supplementary Table 1.

### Body weight and blood glucose level

were measured at two timepoints - just before transplantation and before killing.

### Viability tests

To rule out transplantation failure due to avital islets, viability of isolated islets was demonstrated with dye exclusion test with Trypan blue and Nicotinamide-Adenin-Dinucleotide (NADH)-Diaphorase test [45].

### Proliferative activity

The nucleoside analog 5-Bromo-2`-deoxyuridine (BrdU) (Sigma-Aldrich, Heidelberg, Germany) was applied seven days before killing with an osmotic minipump (Osmotic Pump Model 2001, Charles River Laboratories).

### Tissue processing

Animals were killed under anaesthesia (400 mg/kg bodyweight ketamine, 40 mg/kg bodyweight xylazine) after one or four weeks post-transplantation. Samples of liver tissue and pancreatic tissue were either frozen or perfusion-fixed and embedded in paraffin or fixed in glutaraldehyde and embedded in glycidether. Paraffin slides of 1-2 μm thickness were serially cut and stained with H&E and the periodic acid Schiff (PAS) reaction.

### Enzyme histochemistry

Pieces from frozen liver tissue of two respective WT and chREBP mice were serially cut to 16 μm slices for estimation of the glucose-6-phosphatase (G6Pase, [46]) and Glucose-6-Phosphate-dehydrogenase (G6PDH, [47]) activities.

### Immunohistochemistry

Serial liver sections were stained for Aldolase A, Hexokinase II, Fatty acid synthase (FASN), sterol responsive element binding protein 1 (SREBP1), extracellular related kinase (PanERK), phosphorylated/activated AKT (p-AKT), phosphorylated/activated ribosomal protein S6 (p-RPS6), phosphorylated/inactivated translation repressor protein 1 (p4E-BP1), Transforming growth factor α (TGF α), Epidermal growth factor receptor (EGFR), Insuline receptor substrate 1 (IRS1), mitogen-activated protein kinase kinase 1 (MEK-1), Cyclin D1, cyclin-dependent kinase 4 (CDK4), Tumor necrosis factor α (TNFα), inducible nitric oxide synthase (iNOS), and BrdU (details of applied antibodies are given in Supplementary Table 2). Immunohistochemical signal intensity in CCF was estimated semiquantitatively by comparing CCF with corresponding surrounding extrafocal liver tissue.

Pancreatic tissue serial sections were double stained for BrdU and Insulin (Supplementary Table 2).

### Morphologic and proliferation kinetic investigation

CCF were identified in the liver as lesions of enlarged hepatocytes with pale cytoplasm in H&E staining due to extensive glycogen storage, staining deep purple positive in the PAS reaction (Figures 1 and 2). The corresponding lesions in the enzyme- and immunostained sections were detected by comparison with H&E stained sections. BrdU labeling indices (BrdU-LI) of CCF, extrafocal liver tissue and pancreatic islet β-cells were estimated in representative sections with at least 2000 counted hepatocytes/100 β-cells as percentage of positive stained nuclei per 100 hepatocyte/β-cell nuclei.

### Ultrastructural analysis

Glycidether embedded specimens of 1 mm^3^ liver and pancreatic tissue were cut with diamond knifes with a Leica ultratome to 500 and 750 nm thick semi-thin slides and stained according to Richardson [48]. Ultrathin sections of 70-90 nm were stained with uranyl acetate and lead citrate and examined with a Libra 120 electron microscope from Carl Zeiss (Jena, Germany).

### Statistical analysis

Proliferative activity is expressed based on BrdU-LI. Quantitative data are given as mean ± standard error of the mean (S.E.M.). Differences in body weight, blood glucose level, proliferative activity of CCF were tested using Student`s *t* test. Differences of frequency of CCF were assessed using Fisher`s exact test. Differences were considered significant if p < 0.05.

## CONCLUSIONS

The islet transplantation model is applicable to the mouse, as murine insulin induced hepatocellular CCF resemble preneoplastic liver lesions from other hepatocarcinogenesis models regarding glycogen and lipid storage, as well as increased proliferative activity, which is diminished after knockout of the transcription factor chREBP. chREBP is not only a physiological transcription factor in hepatocytes but rather an essential component of AKT/mTOR mediated cell proliferation and the metabolic switch from a glycogenotic to lipogenic phenotype in early precursor lesions of hepatocarcinogenesis, which will be validated in longterm investigations.

## SUPPLEMENTARY MATERIALS FIGURES AND TABLES


