# Supplementary material for: Hepatocellular glycogenotic foci after combined intraportal pancreatic islet transplantation and knockout of the carbohydrate responsive element binding protein in diabetic mice
Source: Oncotarget. 2017 Nov 1;8(61):104315–29. doi: 10.18632/oncotarget.22234 (PMC5732809; doi:10.18632/oncotarget.22234)
Supplement: Supplementary file 1 [file oncotarget-08-104315-s001.pdf]

Hepatocellular glycogenotic foci after combined intraportal pancreatic islet transplantation and knockout of the carbohydrate responsive element binding protein in diabetic mice

SUPPLEMENTARY MATERIALS

In this study, all animals received humane care according to the criteria outlined in the “Guide for the Care and Use of Laboratory Animals”, prepared by the National Academy of Sciences and published by the National Institutes of Health (NIH publication 86-23 revised 1985). Animal experiments were approved by the Animal Policy and Welfare Committee of the Universitaetsmedizin Greifswald, Germany (LALLF-MV Rostock, Germany, ref. no. 7221.3-1.1-003/12 and 7221.3-1-022/14). Housing of the animals was in accordance with the guidelines of the Society for Laboratory Animal Service and the German Animal Protection Law.

Highly inbred 6-weeks-old male C57BL/6J Wild-type (WT, CHREBP$^{+/+}$) and chREBP-knockout (chREBP-KO, CHREBP$^{-/-}$) mice (n = 297; 25-30 g body weight) were purchased from Charles River Laboratories (Sulzfeld, Germany). Mice were matched to 16 groups (WT/ chREBP-KO, experimental/control, streptozotocine-induced diabetic/not diabetic, one/ four weeks) as shown in Table 3.

Genotyping of B6.129S6-Mlxipl^{tm1Kuy}/J mice

Mouse tails of assumed chREBP knockout (n=55; 0.3 cm length) were incubated in a thermal shaker over night at 55 °C in 300 µl lysis buffer (50 mM KCl, 10 mM Tris-HCL pH 9, 0.45 % Nonidet 40, 0.45 %Tween 20) with 3 µl proteinase K (10 mg/ml). Just before PCR setup the samples were heated to 95 °C for 10 min and centrifuged for 1 min at 21000rpm. 1 µl was applied to the PCR reaction. The separated PCR was performed according to a modified protocol from The Jackson Laboratory (Mlxipl^{tm1Kuy} SEP PCR) on the FlexCycler (Analytik Jena, Germany). In brief, the reaction was carried out using the innuTaq HOT-A DNA Polymerase with 10× PCR Buffer with KCl (Analytik Jena, Germany), in a total volume of 25 µl, containing 0.5 µm of each primer, 200 µm of each dNTP, 2.0 mM $MgCl_2$ and 0.5 U DNA-Polymerase. An initial denaturation for 2 min at 94 °C was followed first by 10 cycles of 94 °C for 20 s, 65 °C for 20 s with -0.5 ° per cycle decrease and 72 °C for 30 s, then by another 28 Cycles of 94 °C for 20 s, 60 °C for 20 s and 72 °C for 30 s with a final denaturation step of 10 min for 72 °C.

Primers were obtained from Eurofins Genomics (Ebersberg, Germany) and sequences were (5’→ 3’) 11421 CGG AGC CAC GCC TCT AA, 11742 GAC CAA CAC CCA ACA CCA G, oIMR4216 CGC CTT CTT GAC GAG TTC. The size of the amplicon for wildtype is 255 bp (primers 11421 and 11742) and for chREBP knockout 450 bp (primers oIMR4216, which targets the neomycin cassette, and 11421).

Diabetes induction

Diabetes was induced with a single intraperitoneal dose of streptozotocin (180 mg/kg bodyweight, Zanosar®, Sigma-Aldrich, Darmstadt, Germany). Mice with an intended blood glucose level > 20 mmol/l after five days were considered diabetic. Transplantation was performed at least after 1 week.

Transplantation

Pancreatic islets were isolated from male donor mice from the same genotype that were killed under anaesthesia and perfused with neutral red solution (Roth, Karlsruhe, Germany). Pancreatic tissue was dissected, reduced mechanically to small pieces and exocrine tissue digested with collagenase/albumin (2.0/1.0 mg per individual pancreas, Serva, Heidelberg, Germany) for 9 minutes. Pure vital islets were collected manually with a pipette under the stereomicroscope and were stored shortly on ice (5-8°C). Recipient WT or chREBP-KO mice received an intraportal transplantation of 60-70 isolated, isologous pancreatic islets into the liver under anesthesia (50-100 mg/kg bodyweight ketamine, 10 mg/kg bodyweight xylazine) via cannulization of the portal vein with a 27 gauge needle, connected with a thin flexible tube system filled with islet particles in 0.1 ml Hanks-solution (Sigma-Aldrich, Darmstadt, Germany).

As intended, the diabetic mice remained hyperglycemic despite islet transplantation, because the number of transplanted islets was low. Continuous hyperglycemia is necessary in this model, so that the islets are stimulated for permanent maximal insulin synthesis and secretion. As a result, local hyperinsulinism and simultaneous hyperglycemia is obtained at the downstream liver acini.

Body weight and blood glucose level were measured at two timepoints - just before transplantation and before killing.

Viability tests

For dye exclusion test with Trypan blue, 100-200 isolated pancreatic islets in Hank`s solution were slowly centrifugated (500 rpm for 5 minutes) and diluted in

Trypan blue solution (0.4 %, 1:1, Thermoscientific, Waltham, MA, USA), incubated for 1-2 minutes in a hemocytometer, and evaluated under the microscope (five times in replicate). Blue stained cells were classified as not viable, unstained cells as viable.

For the Nicotinamide-Adenin-Dinucleotide (NADH)-Diaphorase test, another 100-200 isolated islets were frozen via methylbutane (Roth, Karlsruhe, Germany) in -120°C liquid nitrogen and shortly stored at -80°C (three times in replicate). Cryostat sections of 10µm were stained for enzyme activity of NADH-Diaphorase according to Noguchi et al., 2006 [45]. Cells with blue cytoplasmic staining were considered as viable cells.

Additionaly, islets were stained for insulin (INS anti-mouse antibody, 1:200 overnight, Antikoerper-online ABIN372838), positive reactivity was identified using the Biotinylated universal goat link, Streptavidin AP complex and Vulcan Fast red as chromogen substrate (Biocare Medical, Pacheco, CA, USA).

Modifications of the transplantation procedure

Increase of transplanted islet number

To increase the frequency of CCF in wildtype mice, we conducted transplantation of higher numbers of 120 and 200 isolated islets, mainly in wildtype mice for one and four weeks, and as a control also in one set of chREBP-KO mice (Supplementary Table 1).

Pre-operative insulin treatment

Furthermore, a possible glucolipotoxicity [40] could directly inhibit islet engraftment in portal vein branches immediately after transplantation. In a further additional experimental setting (Supplementary Table 1), diabetic C57Bl/6J wildtype mice received a pre-operative treatment for five days with Neutral Protamin Hagedorn (NPH) Insulin (Protaphane ®, Novo Nordisk, Mainz, Germany; 40IU/kg body weight, subcutaneous [1]), to achieve normoglycemia of 5-16 mmol/l. At the day of transplantation, insulin treatment was discontinued. Diabetic wildtype mice received an intraportal transplantation of 200 isolated islets. Blood glucose level was measured daily prior to transplantation, and 1 day, 3 days and then weekly after transplantation. Control wildtype mice remained without islet transplantation.

Application of the nucleoside analog 5-Bromo-2`-deoxyuridine (BrdU)

Seven days before sacrifice, half of the animals of each group was anaesthetized and osmotic minipumps (Osmotic Pump Model 2001, Charles River Laboratories, Sulzfeld, Germany) filled with 2.5mg of BrdU (Sigma Aldrich, Heidelberg, Germany) were surgically implanted subcutaneously between the scapulae. These pumps continuously delivered BrdU until the animals were sacrificed. The other half of each group received BrdU (8mg/kg bodyweight) 24, 12 and one hour before killing.

Tissue processing

Animals were killed under anesthesia (400 mg/kg bodyweight ketamine, 40 mg/kg bodyweight xylazine) after 1 or 4 weeks post-transplantation. The middle lobe of the liver was clamped, cut into slices and was frozen via methylbutane (Roth, Karlsruhe, Germany) in -120°C liquid nitrogen and stored at -80°C. The remaining liver, pancreas and all other organs were perfused with a fixation mixture of 0.2% glutaraldehyde (Roth, Karlsruhe, Germany), 3% paraformaldehyde (Roth, Karlsruhe, Germany) and 4% Dextran (Roth, Karlsruhe, Germany) in Ringer-solution (B. Braun Melsungen AG, Melsungen, Germany).

The liver was removed and cut into slices of 1-2 mm thickness. Liver lesions of > 1mm and/or transplants were detected and documented under the stereomicroscope. Liver and pancreas tissue was embedded in paraffin or fixed in glutaraldehyde and embedded in glycidether (Serva, Heidelberg, Germany).

Paraffin slides of 1-2 µm thickness were cut and stained by H&E and the periodic acid Schiff (PAS) reaction.

Enzyme histochemistry

Pieces from frozen liver tissue of two WT and chREBP-KO mice were frozen onto the same tissue holder, and serial sections were cut simultaneously in a cryostat (Microm HM 550, Thermoscientific, Waltham, MA, USA), mounted onto the same slide, stained with H&E, PAS and corresponding 16 µm slices for glucose-6-phosphatase activity (lead method [33]) and Glucose-6-Phosphate-dehydrogenase (Tetrazolium salt MTT method [34]).

Immunohistochemistry

Formalin-fixed, paraffin-embedded 1-2 µm thick serial liver sections were manually stained for Aldolase, Hexokinase II, Fatty acid synthase (FASN), sterol responsive element binding protein 1 (SREBP1), extracellular related kinase (PanERK), phosphorylated/activated AKT (p-AKT), phosphorylated/activated ribosomal protein S6 (p-RPS6), phosphorylated/inactivated translation repressor protein (p4E-BP1). Antigen retrieval was performed with a citrate buffer pH 6.0, endogenous peroxidase was quenched with 1% hydrogen peroxide, and positive reactivity of primary antibodies was identified using the HRP polymer and DAB as chromogen substrate (Thermoscientific, Waltham, MA, USA).

Transforming growth factor α (TGF α), Epidermal growth factor receptor (EGFR), Insulin receptor substrate 1 (IRS1), mitogen-activated protein kinase kinase 1 (MEK-1), Cyclin D1, cyclin-dependent kinase 4 (CDK4), Tumor necrosis factor α (TNFα) and inducible nitric

oxide synthase (iNOS) in an automated immunostainer (Leica Biosystems, Wetzlar, Germany) using a DAB (diaminobenzidine) kit (Thermoscientific, Waltham, MA, USA). Immunostaining for BrdU (monoclonal antibody, dilution 1:100 overnight, DAKO, Hamburg, Germany) was performed as described earlier [14, 15].

Immunohistochemical signal intensity in CCF was estimated semi-quantitatively by comparing CCF with corresponding surrounding unaltered liver tissue. Negative controls were stained without a primary antibody. Pancreas BrdU stainings were double-stained with Insulin using an automated immunostainer (Leica Biosystems, Wetzlar, Germany), the UltraVision LP-kit (Thermoscientific, Waltham, MA, USA), and the AP-Polymer and Fast Red as chromogen substrate.

Detailed information about primary antibodies used for immunohistochemistry is listed in Supplementary Table 2.

Morphologic and proliferation kinetic investigations

CCF were identified in the liver as lesions of enlarged hepatocytes with pale cytoplasm in H&E staining due to extensive glycogen storage (positive in the PAS reaction) and were accompanied by lipid droplets in WT mice, but not in chREBP-KO mice. The corresponding lesions in the enzyme- and immunostained sections were detected by comparison with H&E stained sections. Only well-demarcated foci of clear cell phenotype that were easily distinguished from the surrounding liver parenchyma were determined as CCF. BrdU labeling indices of CCF and extrafocal liver tissue were evaluated in representative sections of mice livers containing CCF. If less than 100 hepatocytes were detectable in the CCF, the liver was excluded from the evaluation. Counts of 2000 extrafocal hepatocytes served as internal control. BrdU labeling indices of pancreatic islets were evaluated for all islet cells in one parenchyma section of about 1cm^2, and additionally after double-staining with insulin for β-cells. Levels of proliferation are expressed based on BrdU labeling index (number of positive hepatocyte/total number of hepatocyte nuclei x 100 %, or number of positive islet (or β-) cells/total number of islet (or β-) cell nuclei x 100 %, respectively). Quantitative data are expressed as mean ± standard error of the mean (S.E.M.).

Ultrastructural analysis

Specimens of 2 mm^3 liver containing CCF and pancreatic tissue were cut with a razor blade, fixed in 2.5 % glutaraldehyde, embedded in Glycidether 100, cut with diamond knifes (Science Servcie GmbH, Munich, Germany) with a Leica ultratome Leica EM UC7 (Leica Biosystems, Wetzlar, Germany) to 500 and 750 nm thick semi-thin slides and stained with H&E, PAS and according to Richardson [48]. Ultrathin sections of 70-90 nm were stained with uranyl acetate (Merck, Darmstadt, Germany) and lead citrate (Sigma-Aldrich, Darmstadt, Germany) and examined with a Libra 120 electron microscope from Carl Zeiss (Jena, Germany).

Statistical analysis

Differences in body weight, blood glucose level, proliferative activity of CCF using Student`s *t* test. Differences of frequency of CCF were tested using Fisher`s exact test. Differences were considered significant if $p < 0.05$.

REFERENCES

1. Grant CW, Duclos SK, Moran-Paul CM, Yahalom B, Tirabassi RS, Arreaza-Rubin G, Spain LM, Guberski DL. Development of standardized insulin treatment protocols for spontaneous rodent models of type 1 diabetes. Comp Med. 2012; 62:381-390.

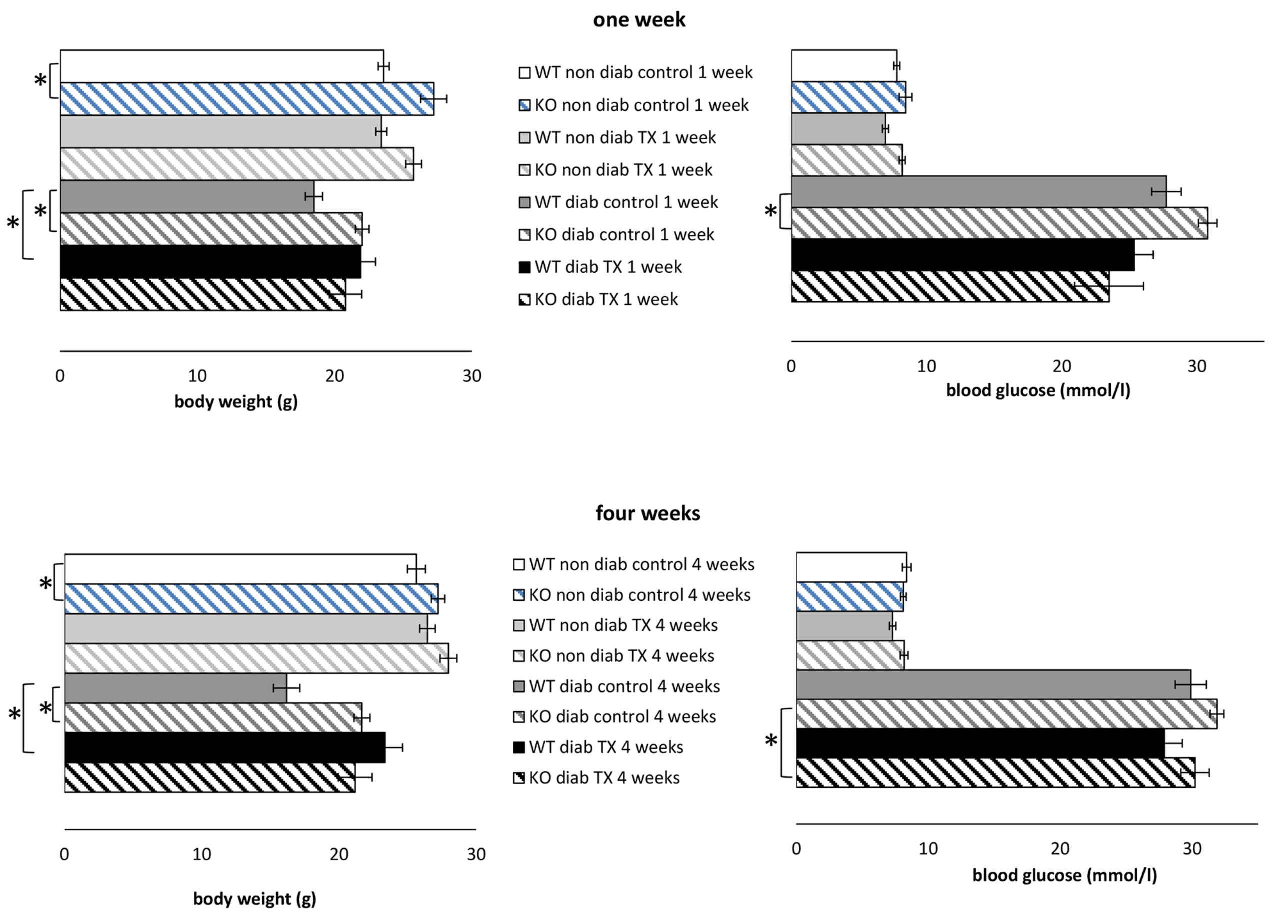

Supplementary Figure 1: Body weight and blood glucose level at the end of the experiment of C57Bl/6J wildtype (WT) and chREBP-knockout (chREBP-KO) mice of all experimental groups (after one and four weeks). TX - after intraportal islet transplantation. mean ± S.E.M.; * $p < 0.05$.

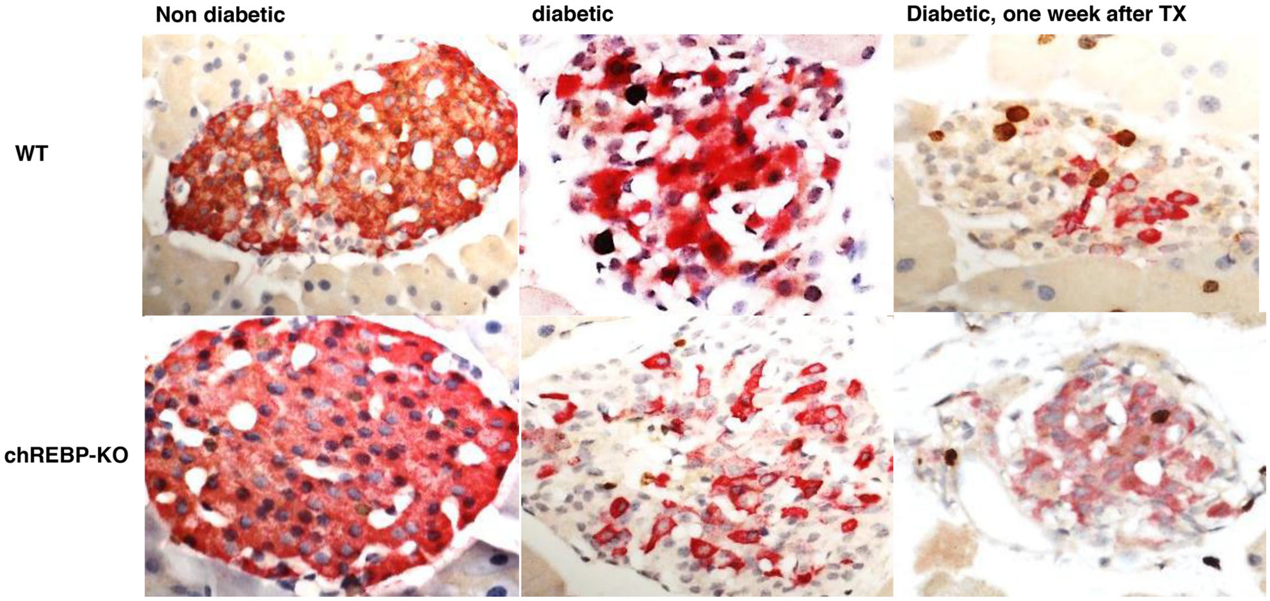

Supplementary Figure 2: Morphology and proliferative activity of β-cells in "in-situ"- intrapancreatic islets in diabetic C57Bl/6J wildtype (WT) and chREBP-knockout (chREBP-KO) mice, one week after intraportal pancreatic islet transplantation (TX) in comparison to controls of diabetic and non-diabetic mice without transplantation. Low proliferative activity in non-diabetic mice without differences between WT and chREBP-KO mice. Reduced insulin positive β-cells (Streptozotocin-induced) in diabetic WT and KO mice with higher BrdU-LI in WT than in chREBP-KO mice. Reduced insulin positive β-cells (Streptozotocin induced) in diabetic WT and chREBP-KO mice, one week after intraportal islet transplantation with slight increased proliferative activity, without difference between genotypes. Please refer also to Table 1. Tissue was double stained with antibodies to BrdU (brown nuclei) and insulin (red cytoplasm). Length of the lower edge: 0.2mm.

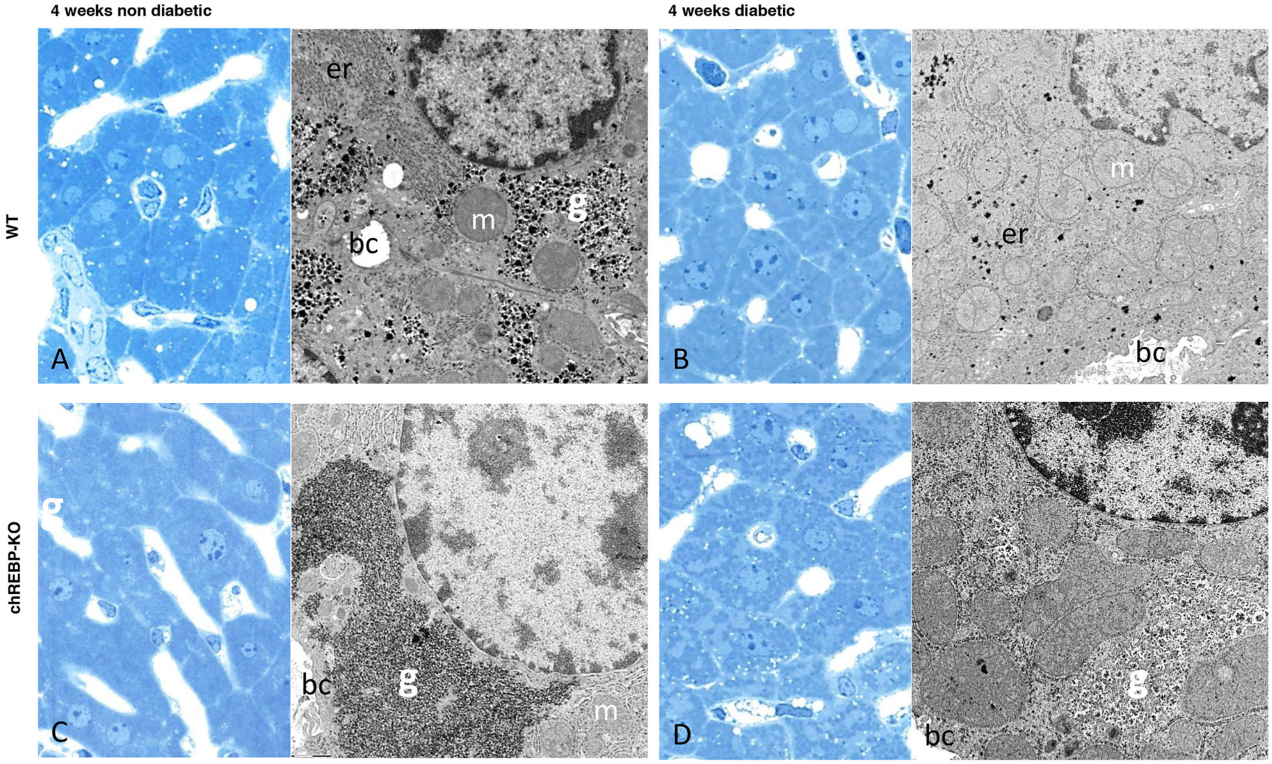

Supplementary Figure 3: Ultrastructure of liver parenchyma of non-diabetic and diabetic mice (control groups, four weeks). Semithin and corresponding ultrathin sections of liver parenchyma of not diabetic and diabetic wild type (WT) and chREBP-knockout (chREBP-KO) mice. **(A)** Not diabetic WT mouse liver with normal hepatocytes, regular content and distribution of glycogen (g) and mitochondria (m). **(B)** Diabetic WT mouse liver showing distinct loss of glycogen and increase of mitochondria (m) and the endoplasmic reticulum (er). **(C)** Non-diabetic chREBP-KO mouse liver containing slight increased glycogen (g) and less mitochondria (m). **(D)** Diabetic chREBP-KO mouse liver comprising retained glycogen α-particles (g) and enlarged mitochondria (m). A-D: bile canaliculus (bc); length of the lower edge: semithin sections 0.06mm, ultrathin sections 10µm.

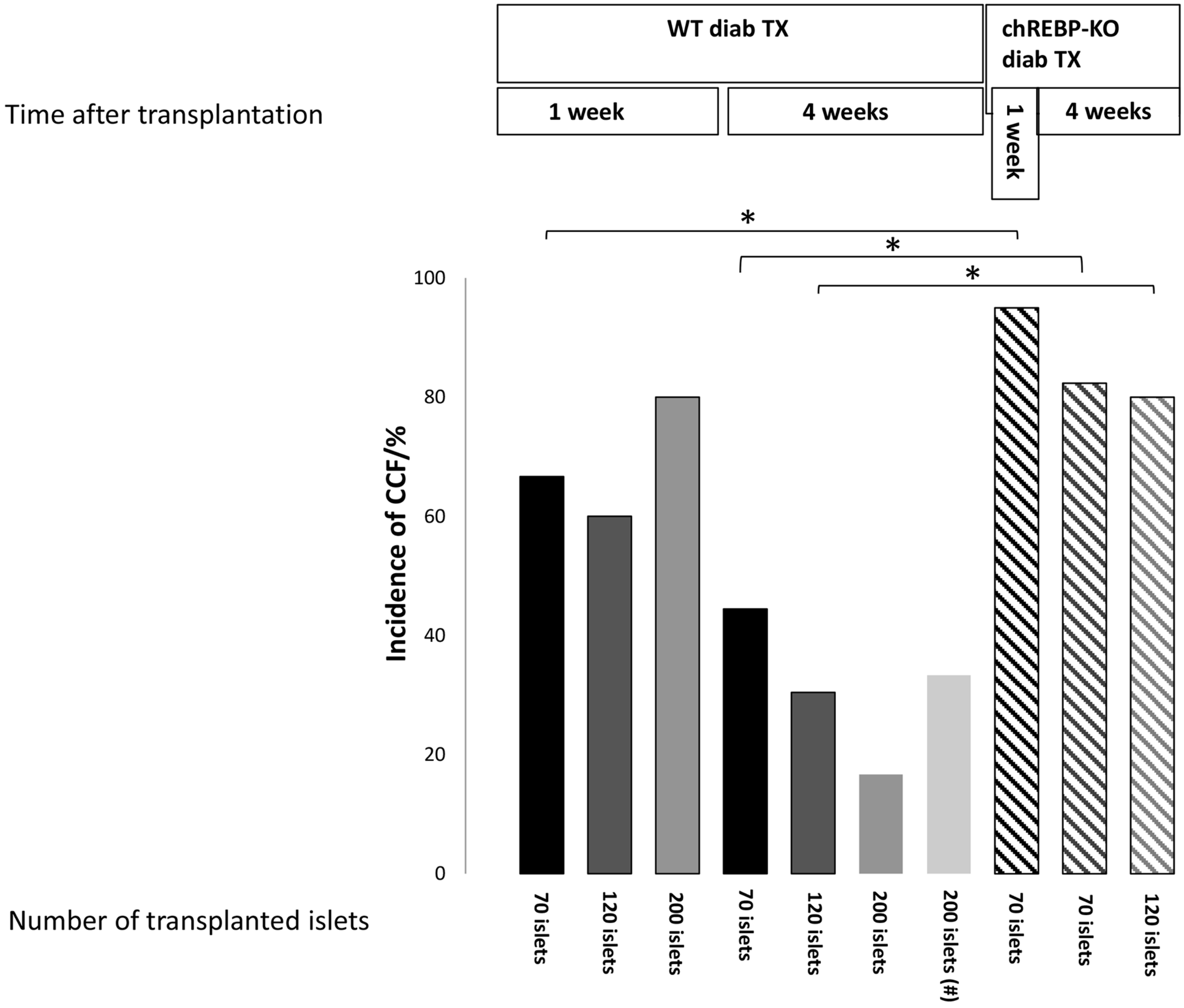

Supplementary Figure 4: Incidence of clear cell foci (CCF) in diabetic wildtype (WT) and chREBP-knockout (chREBP-KO) mice after intraportal transplantation (TX) of isolated pancreatic islets, regarding to time point (1 or 4 weeks) and number of transplanted islets, as indicated. Incidence of CCF is given as % of transplanted mice revealing CCF. * $p < 0.05$ chREBP-KO vs. WT. (#) preoperative NPH insulin treatment (40mg/kg bodyweight, for 5 days).

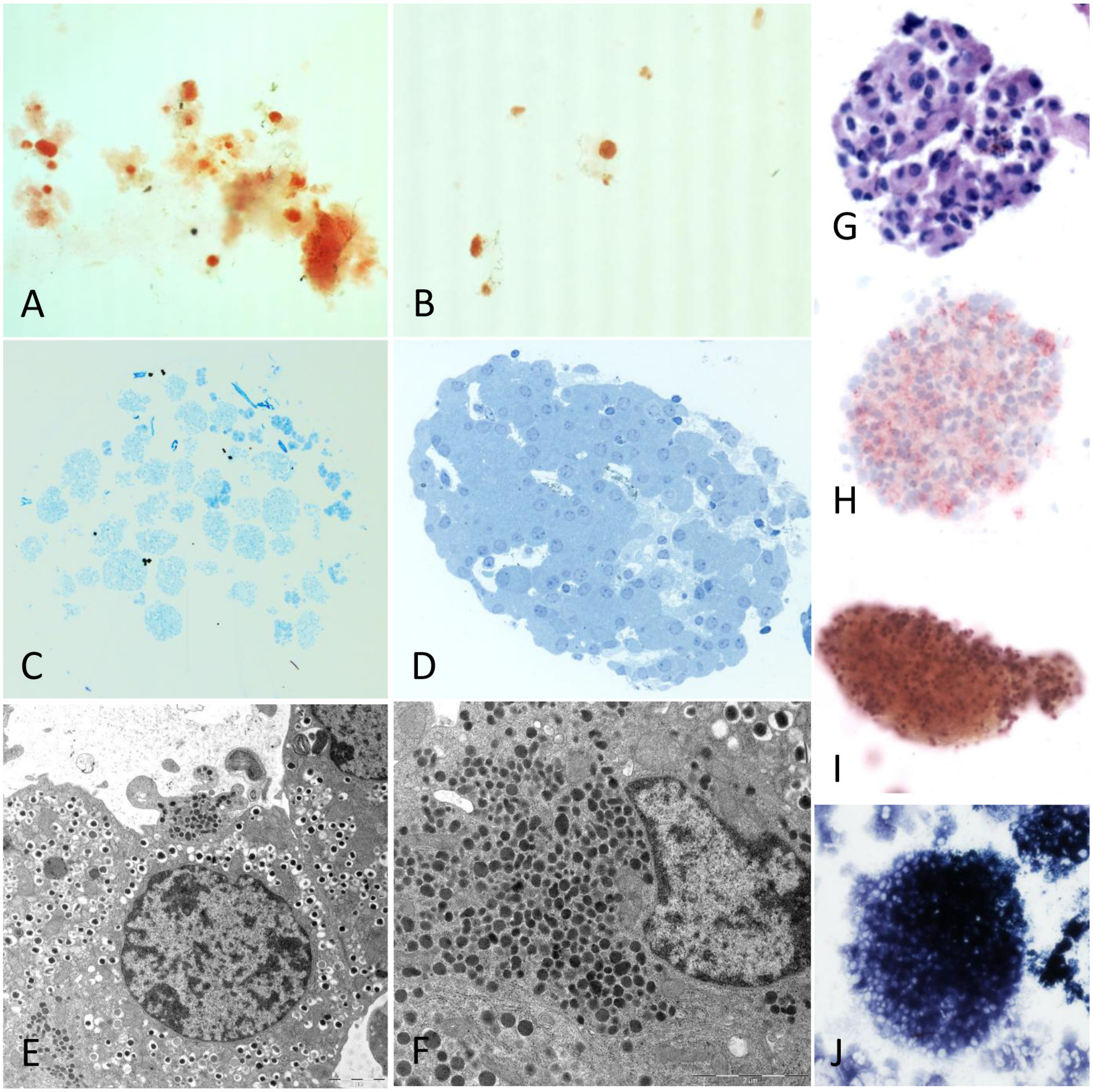

Supplementary Figure 5: Modification experiments: viability of isolated islets. **(A)** Isolated, red stained islets after perfusion with neutral red solution and mechanical reduction to small pieces, with attached exocrine panreas parenchyma. **(B)** Almost pure islets after collagenase digestion prior to transplantation. **(C)** Semithin section of about 20 isolated islets. **(D)** semithin section of one pure islet and corresponding ultrathin sections with vital β-cell **(E)** and α-cell **(F)**. **(G)** Isolated pancreatic islet (H&E); **(H)** immunhistochemical verification of cytoplasmatic insulin expression. Preserved viability in dye exclusion test with Trypan blue without blue staining **(I)** and intact enzyme activity of NADH-Diaphorase **(J)**. Length of the lower edge: A, B, C 2.5mm; D 0.25mm; E, F 9µm; G, H, I, J 0.25mm.

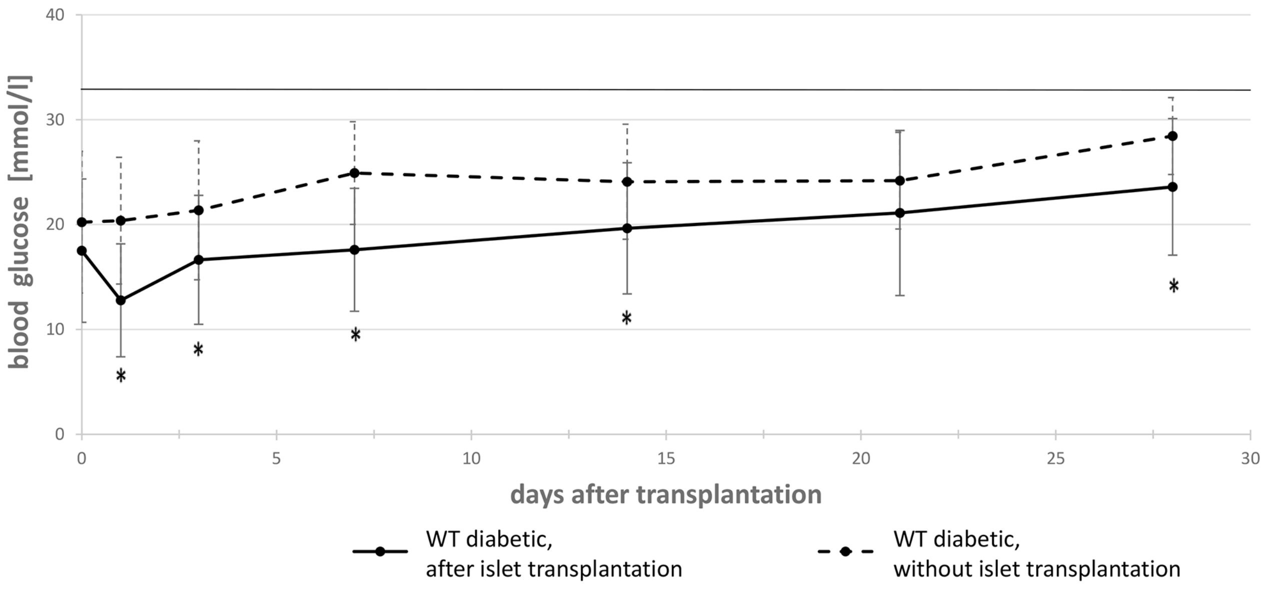

Supplementary Figure 6: Modification experiments: blood glucose level development of diabetic wildtype (WT) mice, after intraportal islet transplantation in comparison to diabetic control mice. Mice were treated with insulin (Neutral-Protamin-Hagedorn, NPH 40mg/kg bodyweight, for 5 days before transplantation). mean ± S.E.M.; * $p < 0.05$.

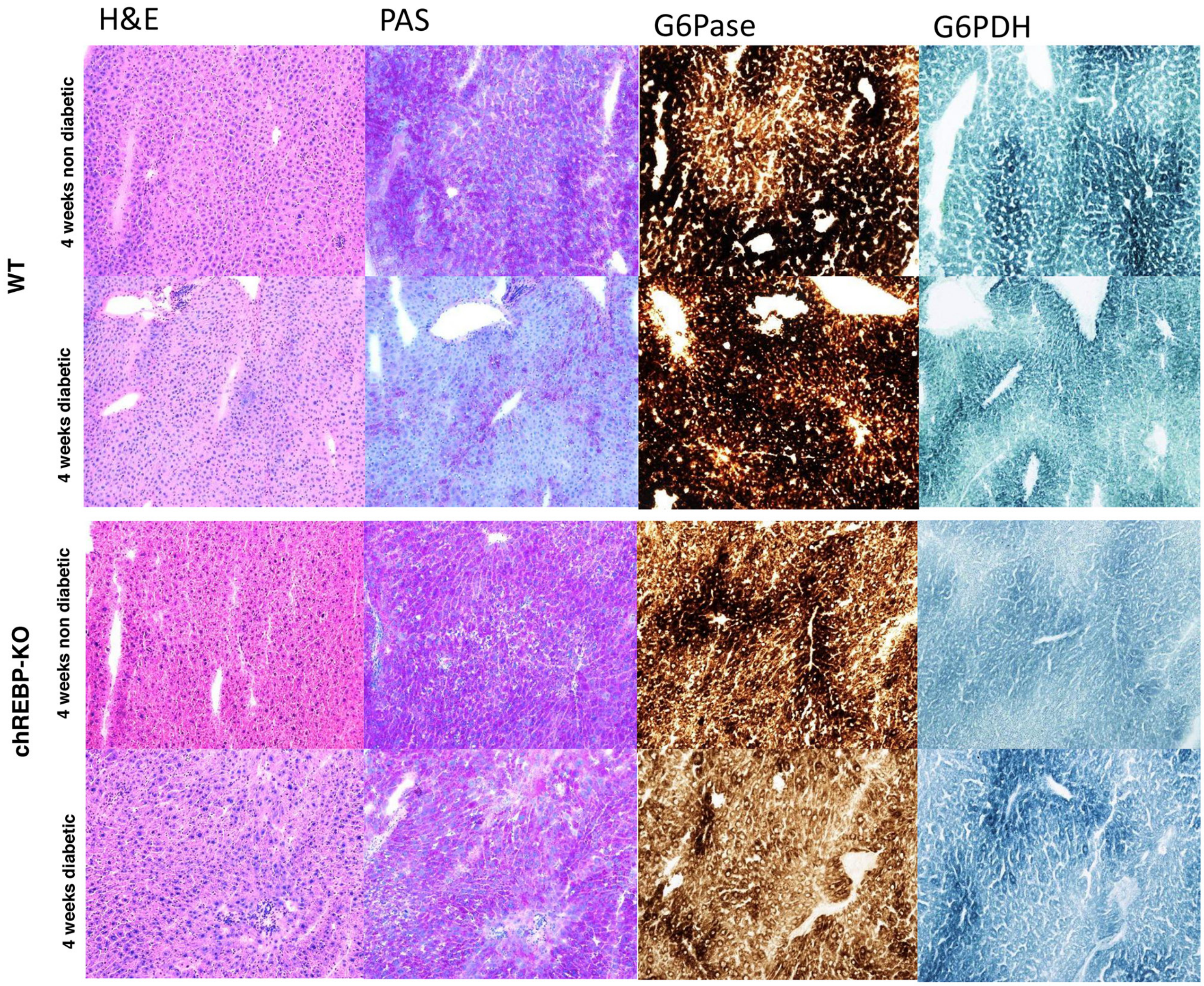

Supplementary Figure 7: Enzyme histochemical findings in control liver tissue of diabetic C57Bl6/J wild type (WT) mice and diabetic chREBP knockout (chREBP-KO) mice after four weeks. Non-diabetic WT mice with regular glycogen storage (PAS) and distribution of the the glucose-6-phosphatase (G6Pase) and glucose-6-phosphate-dehydrogenase enzymes, with a gradient of activity from acinar zone 1 to zone 3. In comparison, non-diabetic chREBP-KO mice contain slightly more glycogen (PAS) and reveal less activity of G6Pase and G6PDH. Diabetic wild type mice show loss of glycogen in hepatocytes (PAS) and a high increase of G6Pase activity and a slight reduction of G6PDH activity, whereas diabetic chREBP-KO mice still contain glycogen (PAS) due to apparent reduction of G6Pase activity. G6PDH activity is elevated in acinus zone 1.

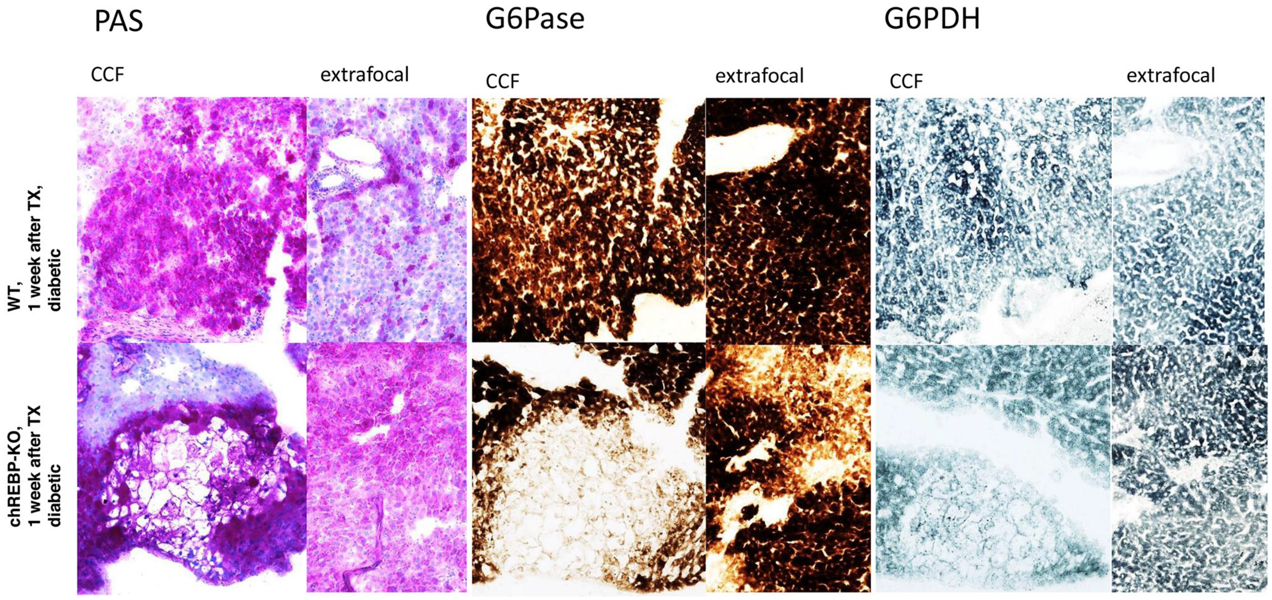

Supplementary Figure 8: Enzyme histochemical findings in clear cell foci (CCF) of altered hepatocytes in diabetic C57Bl6/J wild type (WT) mice and diabetic chREBP knockout (chREBP-KO) mice, one week after intraportal pancreatic islet transplantation (TX). CCF of a diabetic WT mouse downstream of a transplanted islet (*) with increased glycogen storage (PAS) due to less activity of the G6Pase, whereas G6PDH-activity is higher within the focus. CCF of chREBP-KO mouse liver with massive glycogen storage in the PAS reaction, almost complete loss of G6Pase and G6PDH activity. Length of the lower edge: a) 0.9mm, b) 0.6mm.

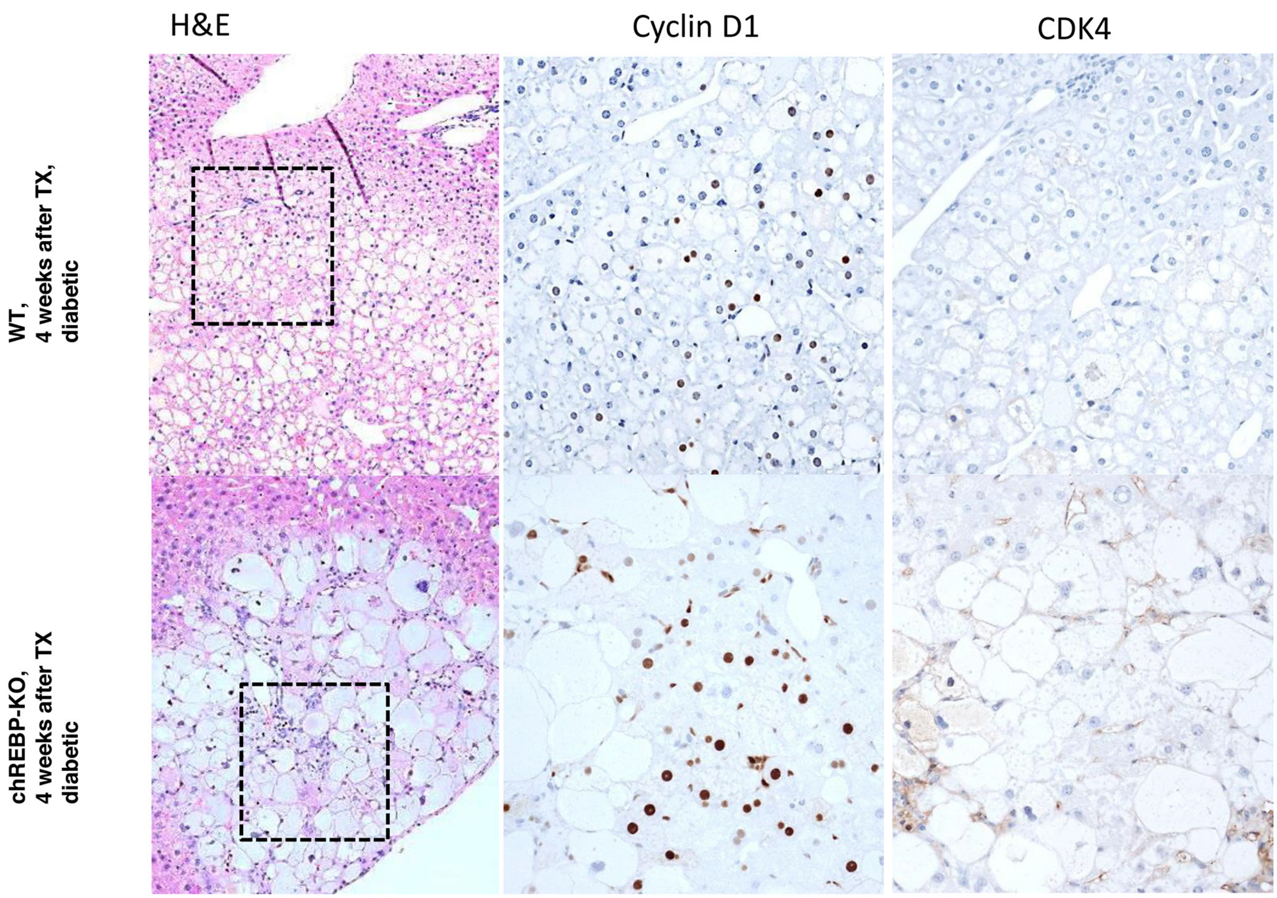

Supplementary Figure 9: Comparison of the expression of Cyclin D1 and CDK 4 in clear cell foci (CCF) of diabetic WT mice and diabetic chREBP-KO mice, four weeks after intraportal pancreatic islet transplantation (TX). CCF (H&E) in WT and chREBP-KO mice with an upregulation of nuclear Cyclin D1 expression without differences between genotypes. CDK4 is not detectable in CCF. Serial paraffin sections. Length of the lower edge: H&E 0.6mm; immunostainings (corresponding to black square in H&E) 0.35mm.

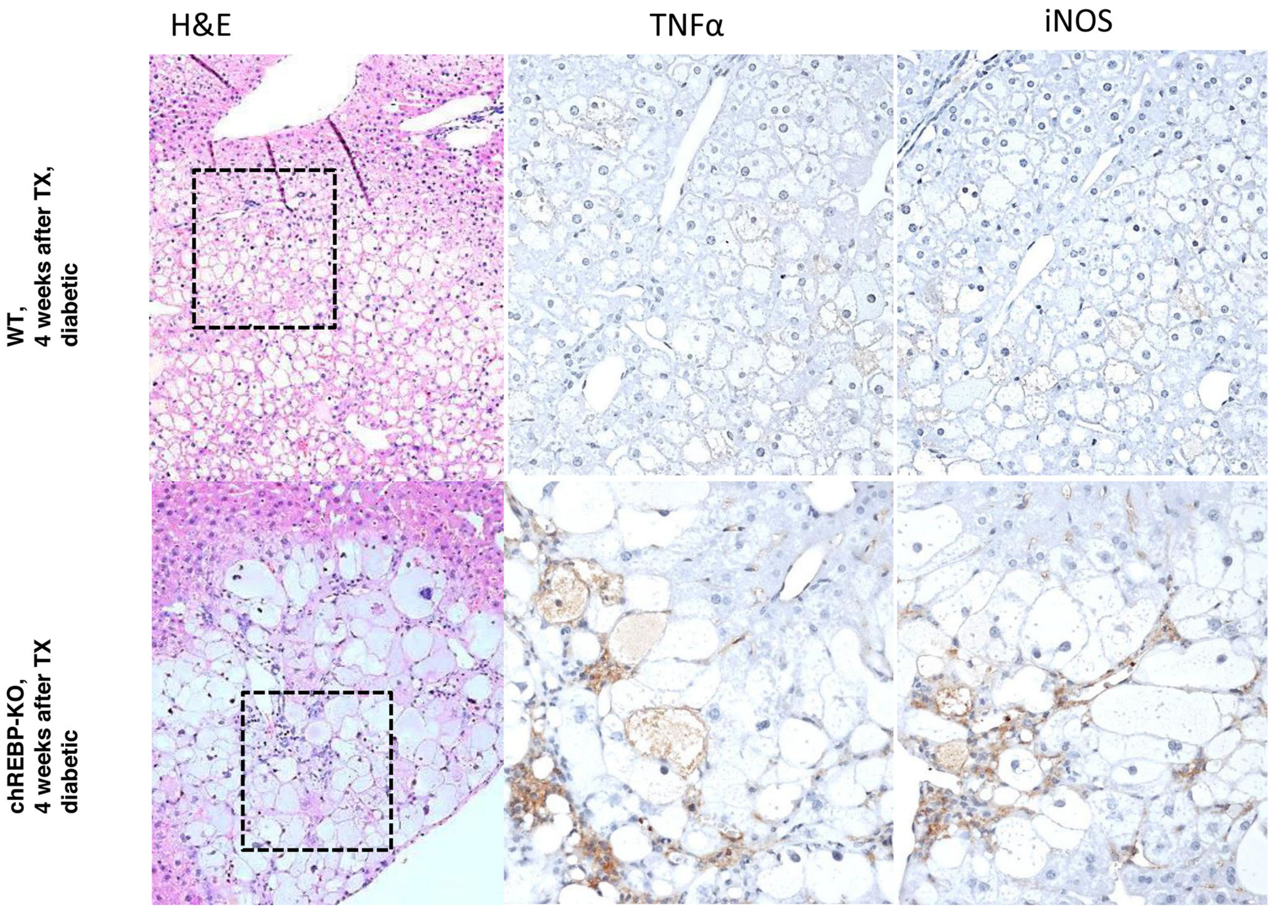

Supplementary Figure 10: Expression of TNFα and iNOS in clear cell foci (CCF) of diabetic chREBP-KO mice, four weeks after intraportal pancreatic islet transplantation (TX). In comparison to WT mice without any inflammatory reaction, CCF (H&E) of chREBP-KO mice reveal an upregulation of TNFα and iNOS in leucocytes between parenchyma cells, but also focally in hepatocytes. Length of the lower edge: H&E 0.6mm; immunostainings (corresponding to black square in H&E) 0.35mm.

Supplementary Table 1: Modification experiments

**Transplanted islets**	**120**	**120**	**200**	**200**		**120**		**200**	**-**
Preoperative NPH inulin treatment	**-**	**-**	**-**	**-**		**-**		**5 days**	**5 days**
	**1 week Diabetic**	**4 weeks diabetic**	**1 week Diabetic**	**4 weeks diabetic**		**4 weeks diabetic**		**4 weeks diabetic**	**4 weeks diabetic**
C57Bl/6J WT	N = 10	N = 23	N = 5	N = 6	chREBP-knockout	N = 20	C57Bl/6J WT	N = 19	N = 16

Supplementary Table 2: List of primary antibodies used for immunohistochemistry in paraffin and cryostat sections

Protein	Dilution	Antibody	
Aldolase A	1/100	rabbit polyclonal	Cell signaling
AMPK α1 α2	1/100	rabbit polyclonal	DAKO
BrdU	1/100 ON	monoclonal	DAKO
CDK4	1/100	rabbit polyclonal	Epitomics
ChREBP	1/25	rabbit polyclonal	Novus Biologicals
Cyclin D1	1/100	rabbit polyclonal	Epitomics
EGFR	1/50	mouse monoclonal	RnD Systems
PanERK	1/100	mouse monoclonal	BD Biosciences
FASN	1/100	mouse monoclonal	BD Biosciences
Hexokinase II	1/300	rabbit polyclonal	Bioss
iNOS	1/100	rabbit polyclonal	Spring Biosciences
Insulin	1/400	rabbit polyclonal	antikoerper-online
Insulin receptor	1/25	rabbit polyclonal	Santa Cruz
IRS1	1/25	rabbit polyclonal	Santa Cruz
p-AKT	1/100	rabbit monoclonal	Cell Signaling
PKM2	1/100 ON	rabbit monoclonal	Cell Signaling
p-RPS6	1/300	rabbit polyclonal	Cell Signaling
p-4EBP1	1/400	rabbit monoclonal	Cell Signaling
SREBP-1	1/100	rabbit polyclonal	Santa Cruz
TGFα	1/100	mouse monoclonal	Merck
TNFα	1/100	gout polyclonal	Santa Cruz

ON: overnight incubation
Antikoerper-online GmbH, Aachen, Germany
BD Biosciences (San Diego, CA, USA)
Bioss Antibodies (Woburn, MA, USA)
Cell Signaling technology Inc (Danvers, MA, USA)
DAKO (Hamburg, Germany)
Epitomics (Burlingame, CA, USA)
Merck, Darmstadt, GermanyNovus Biologicals (Littleton, CO, USA)
RnD Systems (Minneapolis, MN, USA)
Santa Cruz Biotechnology (Santa Cruz, CA, USA)
Spring Biosciences (Pleasanton, CA, USA)
